# *Arthrospira* promotes plant growth and soil properties under high salinity environments

**DOI:** 10.3389/fpls.2023.1293958

**Published:** 2023-12-05

**Authors:** Qiyu Xu, Tao Zhu, Ruifeng Zhao, Yang Zhao, Yangkai Duan, Xiang Liu, Guodong Luan, Ruibo Hu, Sanyuan Tang, Xinrong Ma, Yan Liu, Shengjun Li, Xuefeng Lu

**Affiliations:** ^1^ Key Laboratory of Biofuels, Shandong Provincial Key Laboratory of Energy Genetics, Qingdao Institute of Bioenergy and Bioprocess Technology, Chinese Academy of Sciences, Qingdao, China; ^2^ Shandong Energy Institute, Qingdao, China; ^3^ Qingdao New Energy Shandong Laboratory, Qingdao, China; ^4^ State Key Laboratory of Food Nutrition and Safety, College of Biotechnology, Tianjin University of Science and Technology, Tianjin, China; ^5^ College of Life Science and Technology, Harbin Normal University, Harbin, China; ^6^ State Key Laboratory of Plant Genomics, Institute of Genetics and Developmental Biology, The Innovative Academy of Seed Design, Chinese Academy of Sciences, Beijing, China

**Keywords:** *Arabidopsis*, *Arthrospira* biofertilizer, rhizosphere bacteria, salt stress, soil properties, sweet sorghum, transcriptome

## Abstract

Salt stress detrimentally impacts plant growth, imperiling crop yield and food quality. Ameliorating plant resilience and productivity in saline environments is critical for global food security. Here, we report the positive effect of *Arthrospira* (Spirulina) on plant growth and salt tolerance in *Arabidopsis* and sweet sorghum. *Arthrospira* application greatly promotes seed germination and seedling growth in both species under salt stress conditions in a dosage-dependent manner. Application of 6 mg *Arthrospira* per plate significantly enhances K^+^/Na^+^ equilibrium and reactive oxygen species (ROS) scavenging in *Arabidopsis*, reducing salt-induced toxicity. The primary root length, survival rate, chlorophyll content, photosynthesis, plant height, biomass and yield were all improved in both species. Concurrently, *Arthrospira* demonstrated the synthesis of compatible solutes, such as trehalose (Tre) and glucosylglycerol (GG), contributing to heightened stress tolerance when co-cultivated with *Arabidopsis* on plates. Transcriptome analysis revealed dramatic up-/down- regulation of genes involved in phytohormone signal transduction, chlorophyll and photosynthesis metabolism, and phenylpropanoid metabolism in *Arabidopsis*. Furthermore, the application of *Arthrospira* exerted a positive influence on the rhizosphere bacteriome structure in sweet sorghum, crucial for nutrient cycling and soil health enhancement. Our findings uncovered the underlying mechanisms of algae-plants interaction in saline soil, proposing strategies to enhance crop productivity and soil quality, thereby addressing the urgent need for sustainable agriculture practices to mitigate salinity’s repercussions amidst climate change challenges.

## 1 Introduction

Salt stress is a significant constraint on agriculture (Pan et al., 2021), posing conceivable repercussions for approximately 50% of the world’s arable land by 2050 (Kaashyap et al., 2017). Sodium chloride (Na^+^) primarily causes ion toxicity to plants, resulting in osmotic stress, K^+^/Na^+^ imbalance, which disrupt water and nutrient uptake (Gul et al., 2019; Gadelha et al., 2021). In addition, the imposition of salt stress triggers the synthesis of reactive oxygen species (ROS), instigating potential harm to vital cellular constituents, including DNA, proteins, and lipids. This cascade of events culminates in the attenuation of plant growth and, in severe cases, mortality (Zahra et al., 2022). Salt stress also destroys the structure of chloroplast, photosystem, and electron transfer system, and restricts gas exchange by reducing stomatal opening, which in turn reduces the photosynthetic rate (Negrão et al., 2017; Gadelha et al., 2021). Plants have developed diverse mechanisms to acclimate and endure saline stress conditions, encompassing ion equilibrium, accumulation of osmoprotectants, and orchestration of hormonal regulatory pathways (Ma et al., 2019; Wei et al., 2020).

In recent years, the interaction between plants and microorganisms, such as Rhizobia and Arbuscular Mycorrhizal Fungi (AMF), has emerged as a substantiated contributor to both plant growth and stress resilience (Durán et al., 2018; de Vries et al., 2020). Microorganisms produce amino acids, proteins, metabolisms, thereby furnishing nutrients that contribute to and positively impact the growth of plants (Berendsen et al., 2012; Di Lelio et al., 2023). Furthermore, certain soil microorganisms improve soil quality and ecology by enhancing water and nutrient retention (Abinandan et al., 2019). Additionally, many microorganisms regulate plant metabolism and physiological processes by facilitating nutrient absorption and utilization of N, P, and K, and improving photosynthetic efficiency, leading to significant increase of crop yield (Colla and Rouphael, 2020). Moreover, under stress conditions, microorganisms can accumulate large amounts of osmoregulatory substances, thereby augmenting the plant’s resilience against both biotic and abiotic stressors (Renuka et al., 2018; Abinandan et al., 2019).

Microalgae serve as organic fertilizer in agriculture, reducing chemical fertilizer usage and improving product quality. These biofertilizers play a crucial role in establishing a coherent pathway toward sustainable development (Lu and Valtchev, 2023). Cyanobacteria, which are primitive oxygen-producing photosynthetic organisms, are essential members of rhizobacteria and commonly found in biological soil crust communities. They possess diverse taxonomy and biochemistry, enabling them to synthesize various bioactive molecules like sulfated polysaccharides, amino acids, phenolics, vitamins, phytohormones (auxin, cytokinin, gibberellin, abscisic acid), and compatible solutes. These molecules effectively enhance crop development while conferring resilience against abiotic stressors (Luo et al., 2022). *Arthrospira*, also known as Spirulina, is a salt-tolerant genus of cyanobacteria with applications spanning industries such as health food, wastewater treatment, pharmaceutics, perfumery, and medicine (FAO, 2022; Hamouda et al., 2022). Nonetheless, the understanding of *Arthrospira*-based biofertilizers, especially their mechanisms of action, remains limited.

*Arthrospira* has been proven to improve soil mineral conditions and increase crop yields as a microalgae fertilizer (Álvarez-Gómez et al., 2016; Ismaiel et al., 2022; Pei and Yu, 2022). A recent discovery unveiled a range of physiologically active chemicals in *Arthrospira* extract that could function as plant growth regulators, promoting seedling development and salt stress tolerance in *Triticum aestivum* (Hamouda et al., 2022). *Arthrospira* is also capable of producing intracellular compatible solutes, such as trehalose (Tre) and glucosylglycerol (GG), to adapt to higher extracellular salinity (Warr et al., 1985; Klähn and Hagemann, 2011; Luo et al., 2022). Consequently, the application of *Arthrospira* is expected to impact the composition of rhizosphere bacteria in high-salt environments, highlighting its potential as a sustainable biofertilizer for sustainable agriculture (Rocha et al., 2020).

Despite several reports highlighting the contribution of *Arthrospira* to both plant growth and stress tolerance (Mutale-joan et al., 2021), the comprehensive comprehension of the underlying mechanisms has yet to be achieved. Numerous studies focusing on *Arabidopsis* have elucidated signaling pathways regulating plant growth and stress responses (Zhang et al., 2020; Zhao et al., 2020). Notably, the ABA signaling pathway is pivotal in seed germination, plant development, and senescence, essential for plant survival under adverse conditions like drought and salinity (Liu et al., 2022). The conservative structures and regulatory proteins in *Arabidopsis* establish it as a pivotal model for comprehending the interactions between *Arthrospira* and crops. Sorghum (Sorghum bicolor (L.) Moench) is the fifth most important cereal crop, providing human foods, animal feeds, and bioenergy. Notably, sorghum exhibits exceptional tolerance to abiotic stresses, such as salinity and drought, making it an ideal crop in the face of changing climate conditions (Osman et al., 2022). In this study, by comprehensive analysis of cellular components, we found that *Arthrospira* is capable of synthesizing compatible solutes, among them GG and Tre, and confirmed their positive effect in plant salt tolerance. The transcriptomicc data indicated that *Arthrospira* modifies the expression of genes involved in phytohormone signaling to regulate salt tolerance. Notably, based on 16S rRNA amplicon sequencing and soil nutrient measurements, the *Arthrospira* application also improves soil quality by increasing the abundance of beneficial microbes, water content and soil nutrients. Taken together, this study illuminates the underlying mechanisms of *Arthrospira*-plant interaction under salt stress.

## 2 Materials and methods

### 2.1 Plant materials and growth conditions

The *Arabidopsis thaliana* (Col-0) and *Sorghum bicolor* (M-81E) were used as experimental materials and grown in growth chambers. *Arabidopsis* seeds were subjected to surface sterilization by immersion in a 10% (V/V) NaClO solution for 7 minutes. Subsequently, these seeds were placed onto half-strength Murashige and Skoog (½ MS) medium (pH 5.8), supplemented with 1% (w/v) sucrose and 1% (w/v) agar. Following a 3-day stratification period at 4°C in darkness, the plates were translocated to a growth chamber set at 22°C, operating under long-day conditions (16 hours of light and 8 hours of darkness per photoperiod). The light intensity within the growth chamber was maintained at 90 μmol photons m^−2^ s^−1^. Sorghum seeds were thoroughly surface sterilized and germinated in a growth chamber, maintaining a photoperiod of 16 hours of light (28°C) followed by 8 hours of darkness (23°C). The light intensity in the chamber was set at 600 μmol photons m^−2^ s^−1^. The seedlings of both species were then transferred to either new plates or potted soil with the same growth conditions for subsequent tests after 3-5 days.

In *Arabidopsis*, salt stress treatment was initiated around three weeks post-germination. Each pot was irrigated with 50 mL of a 200 mM NaCl solution every two days. *Arthrospira* treatment commenced one week after the initiation of salt stress. For sweet sorghum, salt stress was induced at the elongation stage, and each pot was irrigated with 1 L of 200 mM NaCl solution every three days. *Arthrospira* treatment was implemented two weeks after the initiation of salt stress.

### 2.2 *Arthrospira* platensis biomass preparation and co-cultivation with plants

Cultivation of *Arthrospira* platensis was conducted under photoautotrophic conditions, utilizing the standard Zarrouk culture medium. The cultivation environment was illuminated with white light at an intensity ranging from 30 to 50 µmol photons m^−2^ s^−1^, while maintaining a temperature of 30°C. *Arthrospira* at the exponential growth stage was filtered with nylon mesh (48 μm pore size), washed with sterilized water, and resuspended in ½ MS medium or tap water in proper concentration as needed. For cell density quantification, a volume of 2 mL from the *Arthrospira* culture was extracted for analysis. The optical density of the cells was quantified at 730 nm (OD730) employing a spectrophotometer (Metash, China). The biomass content of *Arthrospira* was calculated using a calibration curve correlating the OD730 values to biomass fresh weight (FW) concentrations with the following formula: Biomass (g/L) = 7.752*OD_730_ – 1.216 (**Supplementary Figure 1**).

For co-cultivation, the 1/2 MS plates (approximately 20 mL in volume) were inoculated with sterilized water containing various concentrations (1.2, 6, 12, 24 mg FW for *Arabidopsis* and 12, 24, 72, 96, 120, 300 mg FW for sorghum) of *Arthrospira*. For soil treatment, a 1:1 volume ratio of nutrient and regular soil was mixed in a vertical plastic pot. The dimensions of the pots utilized were as follows: 7.4 cm (height) × 6.8 cm (top diameter) × 5 cm (bottom diameter) for *Arabidopsis*, and 31 cm (height) × 33 cm (width) × 23 cm (bottom diameter) for sorghum. *Arthrospira* was sprayed onto the soil surfaces at different concentrations (6, 60, 120, 300 mg FW for *Arabidopsis* and 6, 30 g for sorghum) once a week for three consecutive weeks.

### 2.3 Whole cell component analysis in *Arthrospira*

*Arthrospira’s* crude protein content was assessed through the Kjeldahl method, adhering to the protocol outlined (Barbano et al., 1990). The calculation of Total Kjeldahl Nitrogen (TKN) and crude protein content was achieved by multiplication using a factor of 6.25 (López et al., 2010). The extraction of total lipids was performed utilizing a chloroform and methanol mixture (2:1, v/v), followed by GC-MS analysis, in accordance with our previously established method (Duan et al., 2011). Furthermore, elemental analysis was executed employing Inductively Coupled Plasma Mass Spectrometry (ICP-MS, Shimadzu, Japan) (Dawczynski et al., 2007). The mono- and disaccharides (i.e., glucose, fructose, sucrose, trehalose and glucosylglycerol) were determined by HPLC (High Performance Liquid Chromatography, Shimadzu, Japan) (Lee et al., 2012).

### 2.4 Measurements of physiological and biochemical index

The germination rate and germination-related parameters were determined according to the following equations:


Germination rate (GP, %) =Germinated seeds/Tested seeds × 100%;

Germination index=Σ(Gt/Dt)

Seedling vigor index=GP×(S+L);

The number of germinated seeds (Gt) was recorded along with the corresponding germination days (Dt). Post-germination, the length of the embryonic shoot (S) and the length of the primary root (L) were measured. Measurements were conducted using ImageJ software (Version 1.53t).

The sorghum stem diameter was measured with a caliper using the basal internode 5 cm above the soil surface. Sorghum leaves directly attached to the fourth internode were harvested and their fresh weight was measured utilizing an electronic balance.

Roughly 100 mg of *Arabidopsis* leaves were weighed and subsequently pulverized into a fine powder within liquid nitrogen. The powdered sample was mixed with 1 ml of extraction solution and vortexed for 3-5 minutes. The resultant mixture underwent centrifugation at 8000g and 4°C for 10 minutes, leading to the collection of the supernatant. The levels of hydrogen peroxide (H_2_O_2_) and malondialdehyde (MDA), the enzyme activities of catalase (CAT), superoxide dismutase (SOD), peroxidase (POD) and ascorbate peroxidase (APX) in the leaves, were determined according to manufacturer-specified protocols using PYHA1, PYHA2, PMHA2, PMHA4, PMHA1 and PMHA3, respectively (Molfarming, China).

### 2.5 Measurement of chlorophyll content and fluorescence

The absolute chlorophyll contents of leaves were measured using the spectrophotometer after 95% ethanol extracts, as described previously (Ling et al., 2011). SPAD values were also recorded with a DP-TYS-4N meter (Tuopu, China).

Concomitant chlorophyll fluorescence measurements were conducted employing a MAXI-IMAGING-PAM system (Heinz Walz GmbH, Effeltrich, Germany). To ascertain the maximal fluorescence yield (Fm’), a saturating pulse of red light (1,000 μmol photons m^−2^ s^−1^) was administered. The calculation of the electron transport rate (ETR), indicative of the actual photon flux propelling photosystem II (PSII), was determined using the subsequent equation:


$$ETR=(\frac{Fm^{\prime }-Fs}{Fm^{\prime }})\times \text{ f }\times \text{ I }\times \text{ αleaf}$$

Here, Fs denotes ‘steady-state’ fluorescence, Fm’ represents the maximal fluorescence achieved during a saturating light flash, f signifies the fraction of absorbed quanta utilized by photosystem II (PSII), I stands for the incident photon flux density, and αleaf accounts for leaf absorptance.

### 2.6 Delayed fluorescence analysis

After a 30-minute period of darkness, leaves were positioned on a black cardboard surface prior to image acquisition. Imaging was carried out utilizing the NightSHADE LB985 *in vivo* imaging system, incorporating a CCD camera (Berthold, Germany). The LED channels were configured to wavelengths of 470, 660, and 730 nm, each set at an intensity of 40 μmol photons m^−2^ s^−1^. The measurement of delayed fluorescence extended over a 120-second duration, employing a 4×4-pixel binning. Subsequent data analysis was performed using the indigo software (Berthold, Germany).

### 2.7 Measurement of photosynthetic gas exchange

Photosynthetic gas exchange was evaluated using the CIRAS-3 (PP Systems, Inc., USA) equipped with a 3-cm² leaf chamber. The second fully expanded leaf of each plant was selected for analysis, and parameters including intercellular carbon dioxide concentration, stomatal conductance, and photosynthetic rate were quantified. Illumination was maintained at 2,000 μmol photons m^-2^ s^-1^ of photosynthetically active radiation. The reference CO_2_ concentration was set around 700 μmol CO_2_ mol^-1^ of air (μg mL^-1^). The temperature of the chamber was held constant at 30°C, while the flow rate was maintained at 250 µmol s^-1^. All measurements were executed during a sunny day from 10:00 AM to 2:00 PM.

### 2.8 Leaf sugar determination

Approximately 100 mg of leaf or stem tissues were powdered using liquid nitrogen. Total sugar extraction was performed and quantified through an ion chromatograph (Thermo Fisher, USA), following a previously outlined protocol with minor adaptations (Xu Q, et al., 2018). The separation column employed was a CarboPac PA 10 column, and the mobile phase comprised a 200 mM NaOH solution, running at a flow rate of 1 mL min^-1^. For amperometric detection, a gold electrode served as the working electrode, and an Ag/AgCl electrode functioned as the reference electrode. The elution time spanned 40 min. Standard curves were generated using a combination of three sugars (i.e., Glc, Fru, and Suc). The relative concentrations of these distinct sugars were quantified through the peak area normalization method, executed within the Chromeleon software (Thermo Fisher, USA).

### 2.9 Sodium (Na^+^) and Potassium (K^+^) content analysis

*Arabidopsis* leaves were pulverized using liquid nitrogen. Subsequently, 100 mg of the resulting powder was solubilized in 1 M HCl. The supernatant was collected and diluted with 1 M HCl to an appropriate concentration after filtering the residue (Fang et al., 2018). The quantification of Na^+^ and K^+^ content was executed using a flame photometer (Jingke, China).

### 2.10 Measurement of abscisic acid

*Arabidopsis* leaves were meticulously pulverized into a fine powder using liquid nitrogen. Subsequently, the hormones were extracted utilizing an isopropanol/water/hydrochloric acid solution. This solution was employed to enhance solubility in the organic solvent and to inactivate specific enzymes. The sample was then concentrated through dichloromethane and nitrogen gas blowdown, and the ABA content was measured using HPLC-MS/MS (Shimadzu, Japan; AB SCIEX, USA).

### 2.11 RNA-seq library construction and transcriptome analysis

Total RNA extraction was carried out utilizing Trizol (Invitrogen), adhering to the manufacturer’s stipulations. Evaluation of RNA integrity was performed through employment of the Agilent 2100 Bioanalyzer (Agilent, USA). Subsequently, samples possessing an RNA Integrity Number (RIN) value equal to or greater than 7 were selected for subsequent analyses. Library preparation was undertaken using the TruSeq Stranded mRNA LTSample Prep Kit (Illumina, USA). The transcriptomic sequencing and subsequent analytical procedures were conducted by OE Biotech Company Limited (Shanghai, China).

### 2.12 Rhizosphere soil collection and 16S rRNA sequencing of soil bacteria

After harvesting the sorghum in pots, the non-rhizosphere soils were discarded. Sorghum roots were vigorously shaken to release tightly attached rhizosphere soils and mixed evenly to form a homogenized sample for each plant. DNA extraction from rhizosphere soil samples was executed using the E.Z.N.A.® Soil DNA Kit (Omega, USA). The V1-V9 region of the bacterial 16S rRNA gene underwent PCR amplification using 27F (5’-AGRGTTYGATYMTGGCTCAG-3’) and 1492R (5’-RGYTACCTTGTTACGACTT-3’) primers. Following amplification, SMRTbell libraries were prepared through blunt ligation (Pacific Biosciences, USA). All amplicon sequencing and subsequent analytical procedures were performed by Biozeron Biotechnology Company Limited (Shanghai, China).

### 2.13 Measurements of soil properties

Soil pH was evaluated through the utilization of a soil slurry and a pH meter (METTLER, USA). The traditional gravimetric method was employed to ascertain soil water content (SWC). The concentrations of total nitrogen (TN), Olsen-P, and Avail. K were determined utilizing a soil nutrient meter (YN-4000, China).

### 2.14 Validation via quantitative real-time PCR

For the synthesis of first-strand cDNA, 1 μg of total RNA was employed in conjunction with the PrimeScript RT reagent Kit (TransGen Biotech, China). Quantification of transcript levels in selected genes was performed via qRT-PCR, using the SYBR Green PCR Master Mix kit (TransGen Biotech, China) and the QuantStudio1 real-time detection system (Thermo Fisher, USA), following the manufacturer’s guidelines. The UBQ9 gene was utilized as an internal control for normalization. Three biological replicates of each treatment underwent RNA extraction, and for each sample, three technical replicates were conducted. A comprehensive list of the primers employed is available in **Supplementary Table S1**.

### 2.15 Statistical analysis of data

All outcomes reported in this study were expressed as the mean ± standard deviation (SD). Each experiment was carried out in triplicate. Data analysis was performed using SPSS 26.0 (IBM Corp, Armonk, NY, USA), applying one-way ANOVA (Analysis of Variance) analysis alongside the student’s t-test or the Duncan test as appropriate. Significance levels are denoted as *P<0.05, **P<0.01, and ***P<0.001. In instances where there are no significant differences, letters are sorted alphabetically, commencing from the highest values, with matching letters signifying a lack of significant distinction.

## 3 Results

### 3.1 *Arthrospira* promotes the germination and growth of *Arabidopsis* under salt stress

**Figure 1:**
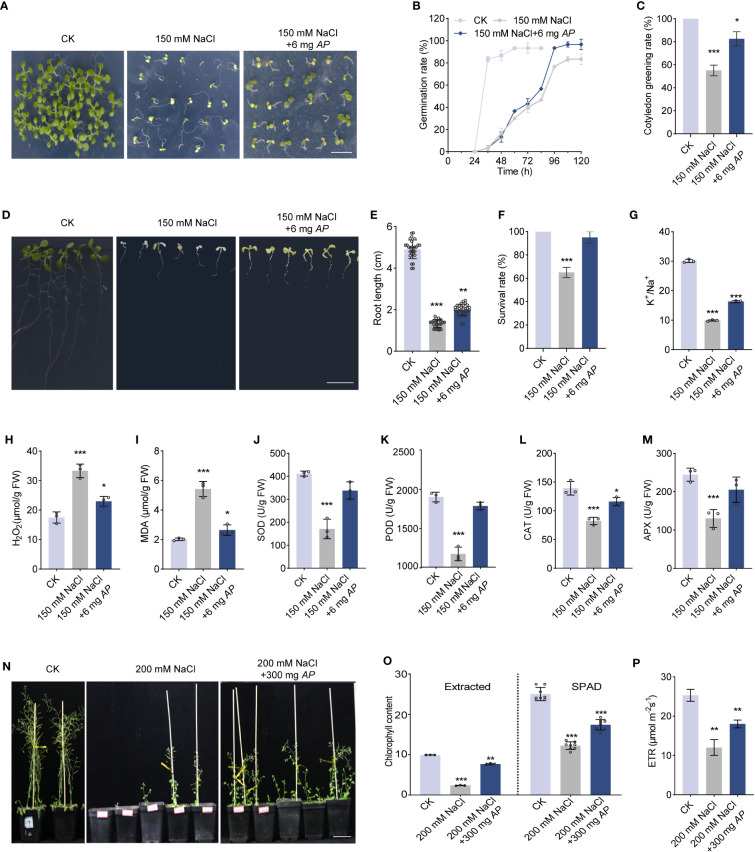
*Arthrospira* improves *Arabidopsis* germination and growth under salt stress. **(A)** Phenotype of 7-day-old *Arabidopsis* cultivated on ½ MS medium with or without 150 mM NaCl containing 6 mg *Arthrospira*. **(B-C)** germination rate **(B)** and cotyledon greening rate **(C)** were compared for seedlings grown in ½ MS medium. **(D)** Phenotypes of 4-day-old *Arabidopsis* seedlings initially grown on ½ MS medium, were subsequently exposed to 150 mM NaCl supplemented with 6 mg *Arthrospira* for another 7 days. **(E-M)** Comparison of root length **(E)**, survival rate **(F)**, K^+^/Na^+^ ratio **(G)**, H_2_O_2_ content **(H)**, MDA content **(I)**, antioxidant enzyme activity for SOD **(J)**, POD **(K)**, CAT **(L)** and APX **(M)** of *Arabidopsis* grown in ½ MS medium. **(N)** Phenotypes of 4-day-old *Arabidopsis* seedlings initially grown on ½ MS medium, were subsequently exposed to soil with 200 mM NaCl for 5 weeks containing 300 mg *Arthrospira*. **(O-P)** Comparison of chlorophyll content **(O)** and relative photosynthetic electron transfer rate (ETR) **(P)** for *Arabidopsis* grown in soil. *AP*, *Arthrospira platensis*. Scar bars, 1 cm in A and D, 5 cm in N. *P<0.05, **P<0.01, and ***P<0.001.

**Figure 2:**
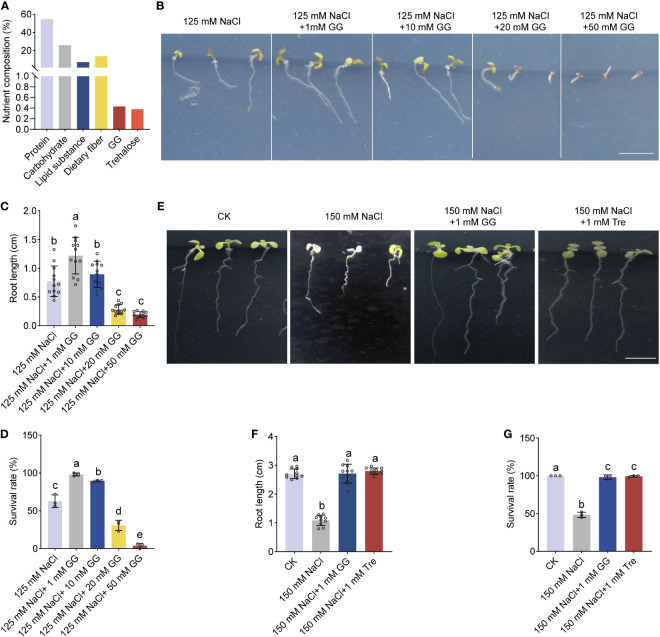
The effect of GG and Tre on seedling growth of *Arabidopsis* under salt stress. **(A)** Comprehensive analysis of the entire cellular components of *Arthrospira*. **(B)** Phenotypes of 4-day-old *Arabidopsis* seedlings initially grown on ½ MS medium, were subsequently exposed to 150 mM NaCl supplemented with various concentration of GG. **(C-D)** Comparison of the root length **(C)** and survival rate **(D)** for *Arabidopsis* grown in ½ MS medium in **(B, E)** P Phenotypes of 4-day-old *Arabidopsis* seedlings initially grown on ½ MS medium, were subsequently exposed to 150 mM NaCl supplemented with 1 mM GG or Tre. **(F, G)** Comparison of root length **(F)** and survival rate **(G)** for *Arabidopsis* grown on ½ MS medium in **(E)** *AP*, *Arthrospira platensis*. GG, Glucosylglycerol. Tre, Trehalose. Scar bars, 1 cm.

To uncover the effect of *Arthrospira* on plant response under salt stress, we supplied various concentrations of *Arthrospira* to the medium containing 150 mM NaCl and investigated the seed germination and seedling growth of *Arabidopsis*. Under salt stress condition, the seed germination was significantly inhibited, while this impact was largely rescued by the application of *Arthrospira* in a dosage-dependent manner, with the optimal concentration of 6 mg FW per plate (**Figures 1A–C**; **Supplementary Figures 2A–E**). The germination, seedling vigor and cotyledon greening rate were all improved upon *Arthrospira* application (**Figures 2C–E**). In addition, the seedling growth, including root length, survival rate and the leaf weight, was dramatically increased after *Arthrospira* application (**Figures 1D–F**; **Supplementary Figures 2F–H**). Furthermore, *Arthrospira* reduced the Na^+^ accumulation and improved K^+^ content in *Arabidopsis* leaves, thus increased the K^+^/Na^+^ ratio and alleviated the ion toxicity caused by salt stress (**Figure 1G**; **Supplementary Figures 2I, J**). Importantly, *Arthrospira* application significantly enhanced the activity of antioxidant enzyme activity, such as SOD (superoxide dismutase), POD (peroxidase), CAT (catalase), and APX (ascorbate peroxidase), which attenuate the toxic effects of H_2_O_2_ and MDA induced by salt stress (**Figures 1H–M**).

Subsequently, we evaluated the effect of *Arthrospira* on plant growth in salt-containing soil. The result showed that plant growth and photosynthetic performance, including chlorophyll content and photosynthetic electron transfer rate (ETR), were significantly improved upon *Arthrospira* application (**Figures 1N–P**; **Supplementary Figures 3A–C**). As soluble sugars in plants are required for salt and osmotic stress response (Saddhe et al., 2021), we examined sugar content using ion chromatography. The content of total sugar, especially sucrose, was increased when subjected to salt stress (**Supplementary Figure 3D**). However, *Arthrospira* suppressed the increment of sugar, suggesting that *Arthrospira* alleviates the damage of salt stress to *Arabidopsis* (**Supplementary Figure 3D**).

### 3.2 *Arthrospira* produces GG and Tre to enhance salt tolerance of *Arabidopsis*

In order to investigate the fundamental mechanism by which *Arthrospira* enhances plant resilience in the face of salt-induced stress, we analyzed the whole cell components of *Arthrospira* grown under 200 mM NaCl conditions (**Supplementary Figure 4**). The result showed that glucosylglycerol (GG) and trehalose (Tre) were synthesized and accumulated, with the amount of 0.43% and 0.38% (dry weight), respectively (**Figure 2A**; **Supplementary Table S2**). GG and Tre belong to the category of compatible solutes, previously documented for their capacity to enhance both plant growth and stress resilience (Tan et al., 2016; Yang et al., 2022). According to the contents of compatible solutes, 6-mg application of *Arthrospira* produces approximately 2.36 mM GG and 1.76 mM Tre, respectively (**Supplementary Table S3**). We next treated *Arabidopsis* with different concentrations of GG and Tre. The result also demonstrated that 1-10 mM GG promotes plant salt tolerance, while a high level of GG inhibits this effect (**Figures 2B–D**). In addition, 1 mM GG and 1 mM Tre showed a significant increment of root growth and survival rate under salt stress conditions (**Figures 2E–G**), supporting their positive promotion in plant salt tolerance.

### 3.3 Transcriptomic analysis by RNA-seq

To better understand the plant’s response on a transcriptional level, we conducted an RNA-seq analysis using *Arabidopsis* seedlings grown in the conditions of normal (CK), 150 mM NaCl with (S_AP) or without (S) 6 mg FW *Arthrospira* (**Supplementary Figure 5A**). After data filtering, each sample generated around 7 Gb of data with an average genome alignment of 98.68% (**Supplementary Table S4**). The principal component analysis (PCA) and correlation analysis showed that all samples were separated into three groups on the PC1 x PC2 score plot with a very strong correlation (**Supplementary Figures 5B, C**).

**Figure 3:**
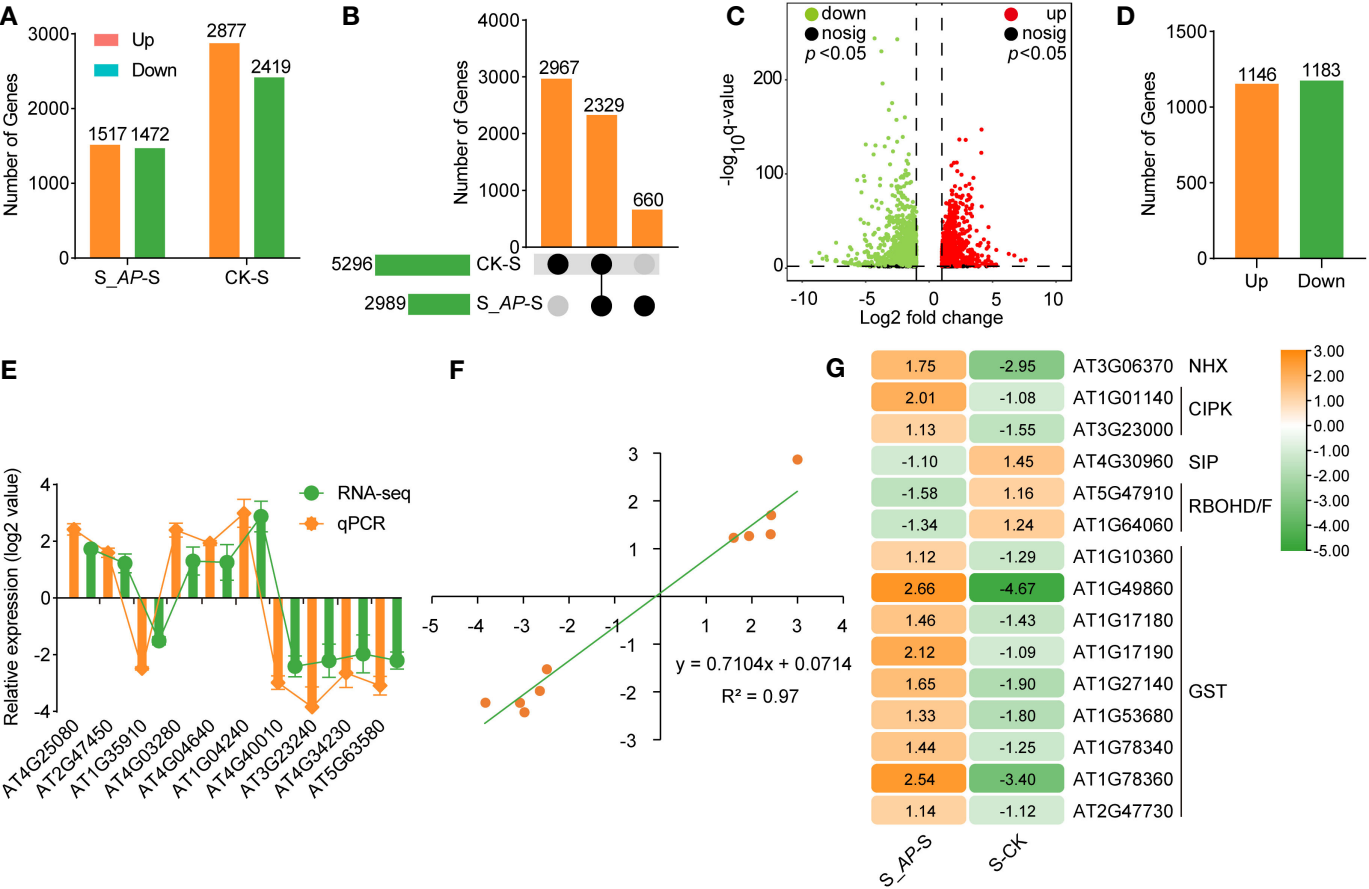
RNA-seq analysis of *Arabidopsis* seedlings cultivated in ½ MS medium with 150 mM NaCl containing 6 mg *Arthrospira*. **(A)** The count of DEGs in S_*AP*-S and CK-S was assessed. **(B)** Upset Venn diagrams of DEGs in S_*AP*-S and CK-S. **(C)** Volcano plot of log_2_-fold change values of shared DEGs between S_*AP*-S and S-CK group. **(D)** Summary of shared DEGs in both S_*AP*-S and CK-S. **(E)** Log_2_-fold change values of selected DEGs in RNA-seq and qRT-PCR. **(F)** Scatter plot demonstrates the consistency of RNA-seq and qRT-PCR data. X axis represents log_2_(S_*AP*/S) and log_2_(CK/S) of RNA-seq, Y axis represents log_2_(S_*AP*/S) and log_2_(CK/S) of qPCR. **(G)** Hierarchical cluster analysis of several classic salt-responsive genes in S_*AP*-S and S-CK. The Orange and green bars or dots indicate up- and down-regulated DEGs in **(A, C, D, G)** respectively. CK represents *Arabidopsis* seedlings grown on normal ½ MS medium condition; S represents *Arabidopsis* seedlings grown on ½ MS medium containing 150 mM NaCl, and S_*AP* represents *Arabidopsis* seedlings grown on ½ MS medium containing 150 mM NaCl and 6 mg *Arthrospira*. AP, *Arthrospira platensis*. DEGs, differentially expressed genes.

Under salt-stressed conditions, 5296 differently expressed genes (DEGs) were identified compared with the normal condition (CK-S). Once applied with *Arthrospira*, 2989 genes were affected (S_AP-S) (**Figure 3A**; **Supplementary Figures 6A, B**). Among the DEGs, 660 and 2967 genes were specifically detected in the comparison groups of S_AP-S and CK-S, respectively (**Figures 3B, C**; **Supplementary Table S5**). Notably, 2,329 overlapped DEGs, including 1,146 up-regulated and 1,183 down-regulated ones, were detected in the two comparison groups (**Figures 3B–D**; **Supplementary Table S5**). To verify RNA-seq data, ten DEGs, including three genes involved in chlorophyll metabolism, two in photosynthesis, three in hormone signal transduction, and two in phenylpropanoid metabolism, were chosen for qRT-PCR validation. The qRT-PCR result exhibited congruence with the RNA-seq results (**Figure 3E**), demonstrating a high correlation coefficient of 0.97 (**Figure 3F**), demonstrating the reproducibility and dependability of the transcriptomic data. Importantly, the expression level of genes required for salt response, including *DREB* (dehydration-responsive element-binding proteins), *LEA* (late embryonic development abundant protein), and *ERD* (early responsive to dehydration), were dramatically affected by salt and *Arthrospira* (**Figure 3G**), demonstrating that *Arthrospira* application recovered the impairment of salt stress by fine-tuning the genome-wide transcriptional level.

### 3.4 *Arthrospira* modifies the signaling pathway of phytohormones

**Figure 4:**
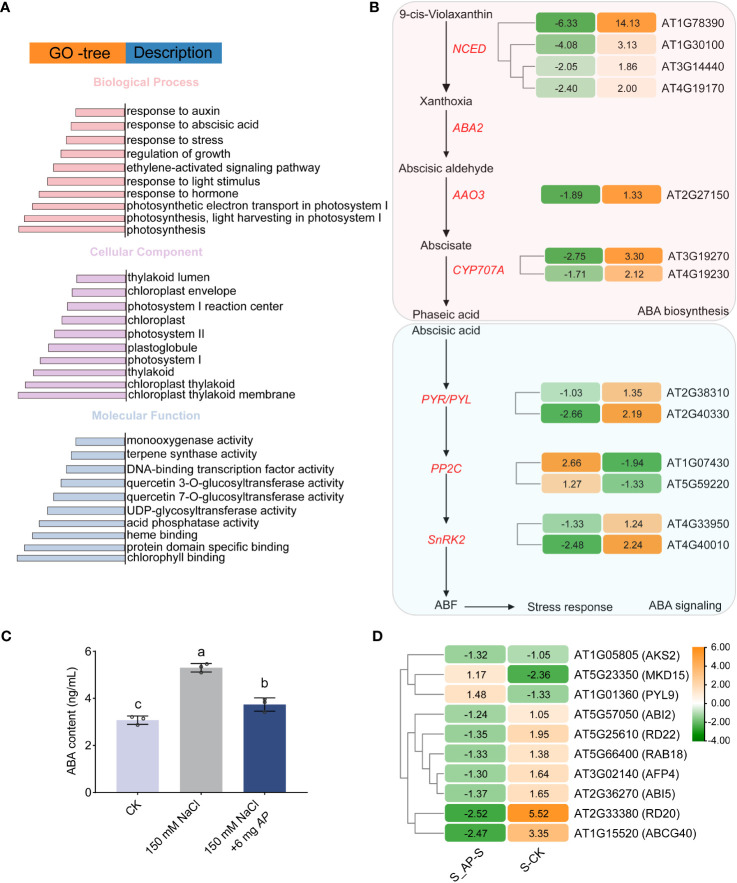
The effect of *Arthrospira* on plant ABA signal transduction pathway of *Arabidopsis* seedlings under salt stress. **(A)** GO enrichment analysis of all shared DEGs. **(B)** Abscisic acid (ABA) biosynthesis and signal transduction cascades in *Arabidopsis* seedlings. **(C)** ABA content in *Arabidopsis* seedlings cultivated in ½ MS medium with 150 mM NaCl with or without 6 mg *Arthrospira*. **(D)** Hierarchical cluster analysis of genes in downstream response of ABA signaling pathway in S_*AP*-S and CK-S. The Orange and green bars indicate up- and down-regulated DEGs in B and D, respectively. Each colored cell represents the log_2_ fold change values. CK represents *Arabidopsis* seedlings cultivated in ½ MS medium condition; S represents *Arabidopsis* seedlings cultivated in ½ MS medium with 150 mM NaCl, and S_*AP* represents *Arabidopsis* seedlings cultivated in ½ MS medium with 150 mM NaCl and 6 mg *Arthrospira*. *AP*, *Arthrospira platensis.* GO, gene ontology. ABA, Abscisic Acid.

Gene Ontology (GO) and Kyoto Encyclopedia of Genes and Genomes (KEGG) enrichment analyses were carried out to elucidate the functional roles of genes and potential signaling pathways associated with salt stress- and *Arthrospira*-induced. Most DEGs were specifically enriched in plant growth regulation, photosynthesis, and phenylpropanoid metabolism (**Figure 4A**; **Supplementary Figure 7**). Importantly, genes required for responses to hormones, including auxin, ABA, and ethylene were enriched (**Figure 4A**; **Supplementary Figure 8**). ABA and ethylene are the important hormones involved in abiotic stress response, while auxin plays a critical role in plant growth (Yu et al., 2019; Waadt et al., 2022). In the RNA-seq data, the expression of genes encoding the rate-limiting enzymes in ABA biosynthesis, 9-cis-epoxy carotenoid dioxygenase (*NCED*), and the cytochrome P450, family 707, subfamily A (*CYP707A*) that involved in ABA hydroxylation were significantly up-regulated upon salt stress (**Figure 4B**). However, once *Arthrospira* was added, their expression levels were significantly reduced (**Figure 4B**). In line with this, endogenous ABA abundance was elevated by salt stress but decreased by *Arthrospira* (**Figure 4C**; **Supplementary Figure 9**). Moreover, the expression patterns of Type 2C protein phosphatases (PP2Cs) and Snf1-Related Kinases 2 (SnRK2s), pivotal constituents of the ABA signaling pathway, were influenced by the presence of *Arthrospira* (**Figure 4B**). The expression level of ABA response genes, such as *RD20, RD29, RAB18 and ABI*5, was reduced under *Arthrospira* treatment (**Figure 4D**). These findings indicate that *Arthrospira* might ameliorate the adverse consequences of elevated salt stress through modulation of the plant phytohormone pathway.

Furthermore, a significant enrichment of genes involved in growth regulation was also observed (**Supplementary Figure 10**). Photosynthesis provides the primary source of organic compounds that are essential for plant growth and fitness. Our data demonstrated that genes required for chlorophyll biosynthesis (Chla and Chlb) were upregulated, while those involved in chlorophyll degradation were significantly downregulated after *Arthrospira* application, leading to increased chlorophyll levels (**Supplementary Figure 11**). Additionally, the genes encoding proteins related to photosystem I (PSI), photosystem II (PSII), antenna proteins, cytochrome b6f complex (PETC), and photosynthetic electron transport showed an increased abundance in *Arthrospira* condition (**Supplementary Figure 11**).

### 3.5 *Arthrospira* modulates gene expression associated with K^+^/Na^+^ homeostasis and ROS elimination

**Figure 5:**
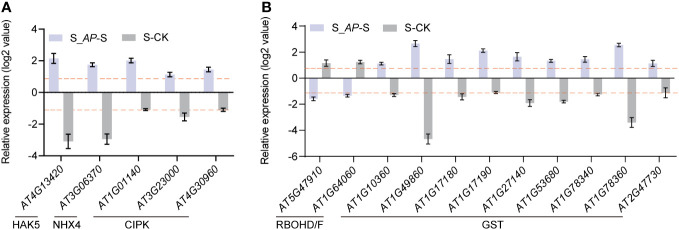
The expression profiles of salt transport- and ROS-related genes. **(A)** Expression levels of HAK5, NHX4 and CIPKs in *Arabidopsis* seedlings analyzed in RNA-seq. **(B)** Expression levels of ROS production genes (RBOHD/F) and ROS scavenging genes (GSTs) in *Arabidopsis* seedlings analyzed in RNA-seq. The log_2_-fold change values in S_*AP*-S and CK-S are presented. CK represents *Arabidopsis* cultivated in ½ MS medium condition; S represents *Arabidopsis* cultivated in ½ MS medium with 150 mM NaCl, and S_*AP* represents *Arabidopsis* cultivated in ½ MS medium with 150 mM NaCl and 6 mg *Arthrospira*.

Since intracellular K^+^/Na^+^ homeostasis is crucial for salt tolerance, we first analyzed the transcriptional level of genes involved in Na^+^ and K^+^ transportation. Based on the RNA-seq data, no notable distinction was observed in the genes implicated in salt overly sensitive (SOS) and high-affinity potassium transporter (HKT). However, the expression of genes regulating Na^+^/H^+^ antiporter (NHX4) and K^+^ transporters (high-affinity K, HAK5) was inhibited by salt stress but induced by *Arthrospira* (**Figure 5A**). In addition, several CBL-interacting protein kinases (CIPKs) associated with the SOS pathway were also increased by the application of *Arthrospira* (**Figure 5A**), implying that the potential regulation of K^+^/Na^+^ homeostasis might involve the modulation of gene expression related to Na^+^ or K^+^ transport.

Moreover, the exogenous addition of *Arthrospira* notably reduced the levels of H_2_O_2_ and MDA in *Arabidopsis* (**Figures 1H, I**), which are crucial components in reactive oxygen species (ROS). The reduced ROS level may result from decreased synthesis or enhanced scavenging. Indeed, the analysis of RNA-seq data revealed that the genes responsible for ROS synthesis, namely RBOHD and RBOHF, were significantly induced, whereas their expression markedly decreased after *Arthrospira* application (**Figure 5B**). Additionally, genes encoding GST subfamily which is required for ROS elimination presented the opposite trend when treated with salt and *Arthrospira* (**Figure 5B**), indicating that *Arthrospira* modified both ROS synthesis and scavenging processes.

### 3.6 *Arthrospira* promotes the germination and growth of sweet sorghum under salt stress

**Figure 6:**
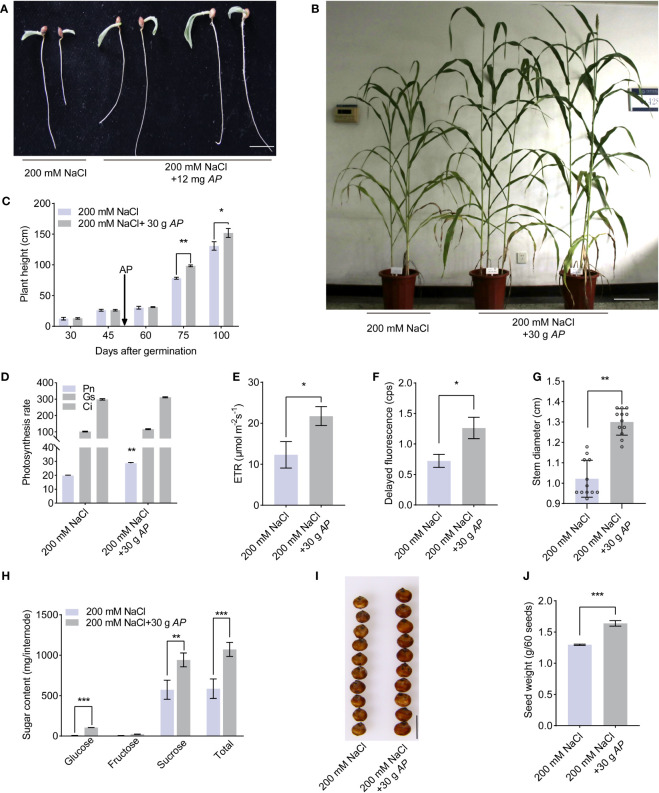
*Arthrospira* treatment improves sweet sorghum germination and growth under salt stress. **(A)** Phenotype of 7-day-old sorghum seedlings grow on filter paper with 200 mM NaCl containing 12 mg *Arthrospira*. **(B)** Phenotype of 100-day-old sorghum grown on soil supplemented with 200 mM NaCl containing 30 g *Arthrospira*. **(C-G)** Comparison of the plant height **(C)**, photosynthesis rate **(D)**, ETR **(E)**, delayed fluorescence **(F)** and stem diameter **(G)** under slat stress with or without 30 g *Arthrospira*. **(H)** Sugar content of sorghum internode. **(I)** Photograph of sweet sorghum seeds under 200 mM NaCl with or without 30 g *Arthrospira*. **(J)** Seed weight of sorghum in **(I)** *AP*, *Arthrospira platensis*. Scar bars, 1 cm in A and H, 10 cm in B. *P<0.05, **P<0.01, and ***P<0.001.

Given *Arthrospira’s* demonstrated amelioration of salt stress impact on *Arabidopsis*, we sought to ascertain its potential in enhancing stress tolerance in sweet sorghum, a globally significant economic crop. To this end, we first compared the germination rate under conditions involving NaCl and *Arthrospira*. Salt stress inhibited the germination speed, germination index, and seedling vigor index (**Figure 6A**; **Supplementary Figures 12A-C**). On the contrary, *Arthrospira* has the ability to decrease the negative effect of salt in a dosage-dependent manner (**Figure 6A**; **Supplementary Figures 12A–C**). Next, we examined the plant performance of soil-grown sweet sorghum to *Arthrospira* application. The result indicated that *Arthrospira* greatly promoted the tolerance and growth of sorghum plants, including plant height, photosynthesis, ETR, and delayed fluorescence (**Figures 6B–F**; **Supplementary Figure 12D**). Most importantly, the important agronomic traits of sweet sorghum, such as stem diameter and total sugar content, were also improved by *Arthrospira* (**Figures 6G, H**; **Supplementary Figure 12E**). The kernel size and weight were also increased up to 20% after the application of *Arthrospira* (**Figures 6I, J**; **Supplementary Figure 12F**). Overall, these findings indicated that *Arthrospira* promotes the germination and growth of sweet sorghum under salt stress, implying its potential application in other crops.

### 3.7 *Arthrospira* affects the structure of rhizosphere bacteriome of sweet sorghum

**Figure 7:**
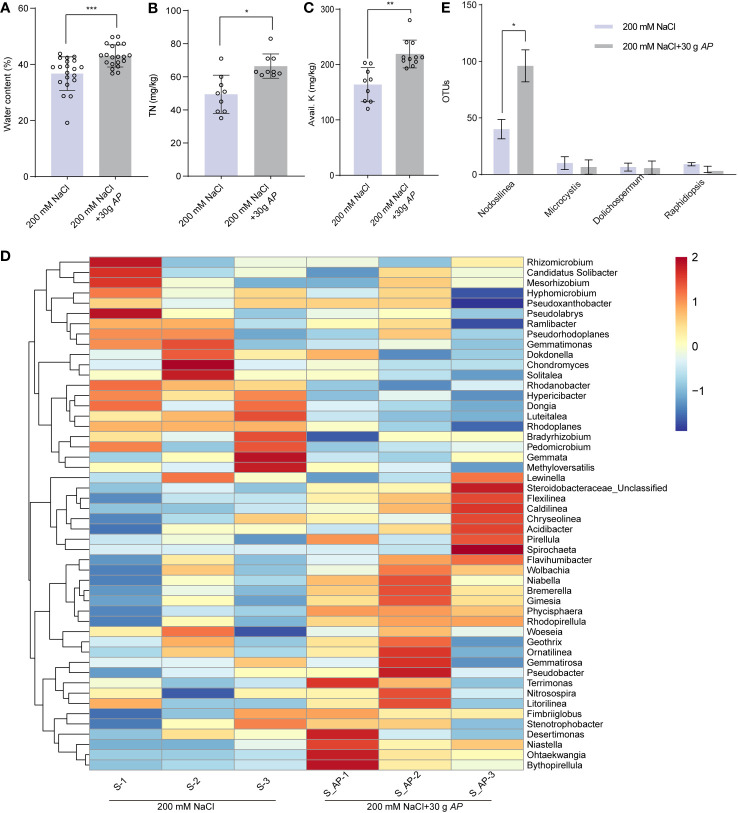
*Arthrospira* alters the soil chemical properties and structure of rhizosphere. **(A-C)** Water content **(A)**, total nitrogen (TN) **(B)**, and Avail. K **(C)** in soil where sorghum grown with or without 30 g *Arthrospira*. **(D)** Heat map displays the genus-level relative abundance of rhizosphere bacterial communities with and without *Arthrospira* treatment. **(E)** Histogram shows the relative abundance of rhizosphere cyanobacteria with and without *Arthrospira* treatment. *P<0.05, **P<0.01, and ***P<0.001.

As mentioned above, *Arthrospira* generates a lot of components which may improve soil quality (**Figure 2A**). To test this, the physical and chemical characteristics of soils grown sorghum with or without *Arthrospira* were examined. The pH and available phosphorus (Olsen-P) showed no significant differences (**Supplementary Figures 13A, B**), likely due to the soil’s inherent weak acidity. However, water content increased by 17.1% (**Figure 7A**), total nitrogen (TN) content increased by 11% (**Figure 7B**), and available potassium (Avail. K) increased by 5% (**Figure 7C**). To investigate whether these changes were related to the rhizosphere community, high-throughput amplicon sequencing of 16S rRNA was performed using rhizosphere soils after sorghum was harvested. A marginal enhancement in the alpha diversity of rhizosphere bacteria was observed with *Arthrospira* application under 200 mM NaCl stress (**Supplementary Table S6**). This was reflected by the Chao1 index, the richness of bacterial communities as well as the ACE index. The prevalent bacterial phyla with relative abundance surpassing 0.5% encompassed Proteobacteria, Bacteroidetes, Planctomycetes, Acidobacteria, Chloroflexi, Actinobacteria, Gemmatimonadetes, Verrucomicrobia, Firmicutes, Spirochaetes, Cyanobacteria, and Armatimonadetes. These phyla collectively constituted 92.3% and 93.4% of the entire rhizosphere bacterial community in the untreated control and *Arthrospira*-treated specimens, respectively. Noteworthy alterations were apparent in the relative distribution of numerous dominant phyla subsequent to the application of *Arthrospira*. For instance, the predominant phylum Proteobacteria reduced from 47.3% to 33.4% and the phylum Acidobacteria decreased from 12.5% to 9.7%. On the contrary, Planctomycetes increased from 7.9% to 15.4% and Bacteroidetes increased from 10.9% to 16.8% (**Supplementary Figures 13C, D**). Following that, an examination was conducted to assess alterations in rhizosphere bacterial communities at the genus level. The findings indicated that the top 50 soil bacterial genera could be generally separated into two major clusters (**Figure 7D**). The upper cluster represents those with a reduced relative abundance after *Arthrospira* application, including *Rhodanobacter*, *Hypericibacter*, *Dongia*, and *Luteitalea* with good repeatability among parallel samples. The lower cluster represents those with increased relative abundance after *Arthrospira* application, including *Steroidobacteraceae*, *Flexilinea*, *Caldilinea*, *Niastella*, *Ohtaekwangia*, and *Bythopirellula* with good repeatability among parallel samples. Compared to that of bulk soil, microbial diversity in the rhizosphere generally was reduced as these environments are with excess available carbon. Many copiotrophs, such as Bacteroidetes and Proteobacteria, are the main phyla that were thought to be enriched (Ling et al., 2022). Our results reveal that the abundance of Proteobacteria and Bacteroidetes not only significantly changed but also in a reverse direction upon *Arthrospira* application in saline soil after sorghum growth, indicating a remarkable perturbation on the beneficial interaction among soil microorganisms, plants, and to some extent, the soil properties. As *Arthrospira* belongs to the phylum Cyanobacteria, then the abundance of rhizosphere cyanobacteria at the genus level was also further compared. *Arthrospira* amplicons were not detected within the bacterial communities of the rhizosphere. The sorghum rhizosphere cyanobacterial genera that dominate under salt stress conditions were identified as *Nodosilinea*, *Microcystis*, *Dolichospermum*, and *Raphidiopsis*. Notably, the application of *Arthrospira* led to a two-fold increase in *Nodosilinea* (**Figure 7E**). Members of this genus are featured by forming nodules along the length of their filament, which are capable of biological nitrogen fixation (Li and Brand, 2007; Perkerson Iii et al., 2011). Taken together, *Arthrospira* application might benefit plants and soil by reshaping the structure of rhizosphere bacteriome which could help to modulate nutrient cycling.

## 4 Discussion

In this study, we report the molecular mechanism and the potential application of *Arthrospira* in plant improvement under salinity stress. *Arthrospira* significantly improves the resistance to salt stress of *Arabidopsis* and sweet sorghum in a dosage dependent manner, whether in the medium or soil. This effect might be due to the biosynthesis of GG and Tre in *Arthrospira* upon salt stress (**Figures 1**, **6**; **Supplementary Figures 2, 3, 12**). Additionally, transcriptomic sequencing revealed that *Arthrospira* modulates the gene expression related to stress adaptation and developmental regulation. Most importantly, *Arthrospira* application significantly modifies the rhizosphere structure, thereby improving soil quality, which could provide an approach to develop a sustainable agriculture in the world.

### 4.1 *Arthrospira*-mediated regulation of ion homeostasis enhances plant salt tolerance

During salt stress conditions, preserving plant morphology and yield hinges upon a range of physiological determinants and intrinsic regulatory mechanisms, encompassing ROS scavenging, ion transportation, and hormone homeostasis (Zhu, 2016; Zhao et al., 2020). The physiological stability of plants relies to a great extent on the dynamic balance of Na^+^ and K^+^ within their cells (Jiang et al., 2012). High levels of Na^+^ inhibit K^+^ absorption, disrupt ion equilibrium, and hinder plant growth. In this investigation, the application of *Arthrospira* resulted in a noteworthy reduction in Na^+^ content while concurrently elevating K^+^ levels, thus maintained K^+^/Na^+^ homeostasis under salt stress (**Figure 1**; **Supplementary Figure 2**). RNA-seq analysis and endogenous ion measurements revealed that *Arthrospira* application significantly upregulated the expression of *HAK5* (AT4G13420) and *NHX4* (AT3G06370) under salt stress, as well as CIPK kinases associated with the SOS pathway (**Figure 5**). *NHX4* encodes a Na^+^/H^+^ antiporter accountable for compartmentalizing Na^+^ from cytoplasm into the vacuole (Wang et al., 2016), the expression pattern indicating that application of *Arthrospira* could enhance the Na^+^ compartmentalization and promotes vacuolar sequestration. HAK5 mediates K^+^ absorption through the plasma membrane and maintains K^+^/Na^+^ homeostasis under salt stress (Jung et al., 2009; Nieves-Cordones et al., 2019). Besides, CIPK9 (AT1G01140) and CIPK6 (AT4G30960, SIP3) were reported to interact with SOS2 and SOS3, respectively, to modulate K^+^ homeostasis (Guo et al., 2001; Pandey et al., 2007). Furthermore, CIPK6 has the capacity to directly regulate the potassium channel AKT2 (Held et al., 2011), while CIPK9 interacts with HAK5 to regulate K^+^ uptake (Lara et al., 2020). These observations imply that *Arthrospira* exerts stronger regulatory effects on K^+^ compared to Na^+^, enhancing the control of K^+^/Na^+^ ratios during salt stress and mitigating ion toxicity in plants by fine-tuning genes involved in ion homeostasis, thus enhancing salt tolerance.

### 4.2 *Arthrospira* mitigates oxidative stress and ion imbalance for improved plant salt resilience

Secondary stress induced by salt stress, particularly oxidative stress, significantly inhibits plant development. Salt stress triggers the outbreak of ROS within plant cells, leading to detrimental oxidative conditions that damage cell membrane, cause lipid peroxidation, and generate aldehyde compounds like malondialdehyde (MDA) (Wang et al., 2008; Olsen et al., 2013). The balance between ROS production and clearance system largely regulates their action within plant cells. In comparison to the salt stress group, the addition of *Arthrospira* was found to decrease H_2_O_2_ and MDA level while increasing the activity of multiple antioxidant enzymes (**Figure 1**), indicating a potential modulation of genes involved in ROS homeostasis. This was further confirmed through RNA-seq analysis of gene expression related to ROS and antioxidant enzyme activities. *Arthrospira* supplementation significantly reduced the expression of ROS synthesis-related gene *RBOHD/F* (**Figure 5**). Although multiple antioxidant enzyme activities were significantly affected, only GST-like genes showed significant enrichment in the RNA-seq analysis (**Figure 5**). Moreover, overexpressing of poplar *HAK6*, the homologous gene of *HAKA5* in *Arabidopsis*, enhanced K^+^/Na^+^ control during salt stress and promoted maintenance of ROS homeostasis (Xu et al., 2023). Therefore, we propose that exogenous *Arthrospira* facilitates the maintenance of ROS balance within plants during salt stress through the dual effects of controlling K^+^/Na^+^ levels and enhancing antioxidant enzyme activities.

### 4.3 *Arthrospira’s* multi-pathway regulation: elevating salt tolerance and growth harmony in plants

*Arthrospira* hydrolysates have emerged as promising biostimulants for various plant species, enhancing growth, photosynthetic efficiency and yield (Santini et al., 2022; Shedeed et al., 2022). In this study, the presence of compatible substances like GG and Tre in the lysate was suggestive of their potential pivotal contribution in enhancing plant salt tolerance and promoting growth (**Figure 2**). Previous studies have shown that exogenously Tre influences downstream gene expression of ABA signaling, thereby modulating salt tolerance in tomato plants without affecting ABA synthesis (Yu et al., 2019). Our investigation further revealed that the application of *Arthrospira* not only altered endogenous ABA levels but also regulated downstream gene expression under salt stress conditions (**Figure 4**), highlighting the complex effects imparted by *Arthrospira* supplementation. Notably, under salt stress, *Arthrospira*-treated plants exhibited reduced ABA content compared to the control, selectively repressing AtNCED while promoting upregulation of CYP707A (**Figure 4**), indicating *Arthrospira’s* potential role in negatively regulating ABA metabolism, consistent with prior studies in soybean and apple (Li et al., 2015; Asaf et al., 2017). The significant transcriptional upregulation of SnRK2s, crucial in ABA signaling, along with downstream gene expression (**Figure 4**), suggests that *Arthrospira* treatment actively stimulates the ABA signaling pathway, potentially initiating stomatal regulation in response to salt stress, as observed in tomato and *Arabidopsis* transgenic plants (Sun et al., 2011; Yu et al., 2019). CIPK6, previously identified as a kinase regulating ion homeostasis, is also involved in the ABA pathway by mediating ABA signal transduction and influencing downstream gene expression (Chen et al., 2013). Therefore, precise regulation of ABA level and downstream signaling is of great significance for simultaneously enhancing salt tolerance and growth. It is pertinent to acknowledge that the regulation of growth may also be achieved through comprehensive control of auxin, chloroplasts, and photosynthesis (**Supplementary Figures 8, 10, 11**). Moreover, although the biosynthetic pathway for GG is absent in higher plants, studies have successfully transformed *Arabidopsis* and potato with *ggpPS* gene from the γ-proteobacterium *Azotobacter vinelandii*, encoding a combined GG-phosphate synthase/phosphatase (Klähn et al., 2009; Sievers et al., 2013). It has been observed that low concentrations of GG in *Arabidopsis* leaves enhance salt tolerance without impacting growth, while moderate or high levels of GG inhibited plant growth without improving salt tolerance (Klähn et al., 2009). This aligns with our findings that GG exhibits a dosage-dependent effect on plant salt stress and growth (**Figure 2**). Despite previous research have highlighted the significant role of Tre in processes like ABA and ROS regulation, the involvement of GG in these mechanisms has not been investigated. Our study provides additional insights into the essential functions of both compounds. While Tre and GG exhibited notable enhancements in plant salt tolerance in agar plates, the impracticality of their direct application to soil-grown plants arises from the high cost of extraction and purification from *Arthrospira*. Nevertheless, *Arthrospira* holds promise for application in farm fields, particularly those with high salinity, to enhance crop tolerance and improve soil quality. Collectively, these findings suggest that the addition of *Arthrospira* may achieve a better balance between improving resistance and promoting growth by finely regulating multiple pathways.

### 4.4 *Arthrospira* enhances soil and rhizobacteria for improved plant growth and nutrient dynamics

*Arthrospira* addition significantly improved soil properties, nutrient status (**Figure 7**), and root-associated bacteria, known for their beneficial role in nutrient acquisition and plant pathogen defense. Under salt stress or with the addition of chemical fertilizer (Wu et al., 2021; Yukun et al., 2021), the alpha diversity of rhizosphere bacteria in sorghum significantly decreases. However, in this study, we observed that the bacterial richness within the rhizosphere soil under 200 mM NaCl stress exhibited no noteworthy distinction when compared to the *Arthrospira*-treated soil under analogous circumstances (**Supplementary Table S6**). This suggests that *Arthrospira* treatment can enhance soil properties and crop yield by maintaining the richness of rhizobacteria. Although the assembly patterns of the rhizosphere bacterial community vary among plant species, they can exhibit great similarities within the same plant species even in different environments (Xu J, et al., 2018; Trivedi et al., 2020). Our taxonomic analysis revealed that the dominant phyla of sorghum rhizobacteria are broadly consistent with findings from other studies (**Supplementary Figures 13C, D**) (Yukun et al., 2021). Proteobacteria, the most abundant phylum, tends to be more abundant in high-salinity soil (Yukun et al., 2021). Intriguingly, we found *Arthrospira* treatment significantly reduces the abundance of Proteobacteria under salt-stress conditions (**Supplementary Figures 13C, D**). This beneficial alteration indicates *Arthrospira* application could lead to fundamental changes in soil microbial-driven functions. The increased relative abundances of Planctomycetes and Bacteroidetes (Ravinath et al., 2022), two phyla of representative plant growth-promoting bacteria, demonstrates the important role of *Arthrospira* application on sorghum performance from another perspective, beyond salt tolerance. Certain Planctomycetes, such as *Pirellula*, have demonstrated the capability to enhance plant growth by generating hydrogen sulfide (H_2_S), a factor that exerts a favorable influence on root development. Additionally, this process is conducive to ABA-mediated responses to abiotic stress, notably by instigating stomatal closure (Ravinath et al., 2022). This finding aligns with our transcriptome analysis of *Arabidopsis* (**Figure 5A**). Additionally, we did observe the enrichment of Pirellula at the genus level (**Figure 7D**). The bacteroidetes genus *Niastella* possesses phosphate solubilization trait through its alkaline phosphatase activity, and its relative abundance also obviously increased (**Figure 7D**). While *Arthrospira* is unlikely to colonize the sorghum rhizosphere, it enhances the abundance of *Nodosilinea*, likely to contributing to nitrogen fixation and regulating sulfur metabolism (Perkerson Iii et al., 2011; Carrión et al., 2017).

**Figure 8:**
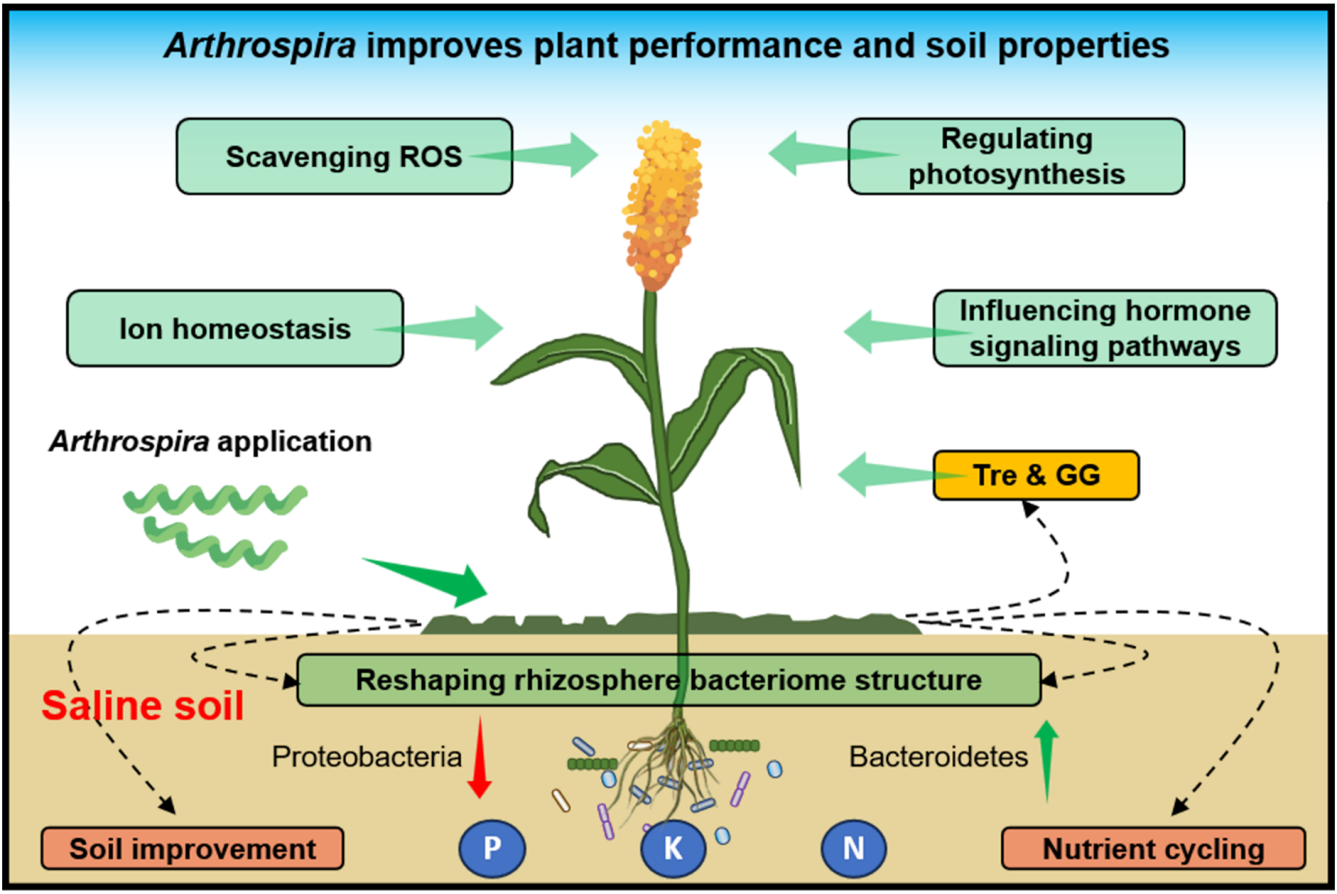
Schematic model of *Arthrospira* promotes plant growth and soil properties under high salinity environments. Shown *Arthrospira’s* positive impact on plant growth, soil enhancement under salt stress, achieved through mechanisms like ion balance, enhanced antioxidants, improved photosynthesis, hormone modulation, and microbial influence, positions it as a promising biofertilizer for sustainable agriculture in saline environments.

Overall, this study highlights *Arthrospira’s* beneficial impact on plant growth and soil enhancement under salt stress. Mechanisms encompass: (i) Reducing sodium accumulation, fostering potassium uptake for ion balance; (ii) Boosting plant antioxidants, mitigating ROS-induced stress; (iii) Enhancing photosynthesis efficiency via gene alteration, augmenting energy and nutrient production; (iv) Modulating plant hormones (e.g., ABA, auxin), regulating stomatal closure and stress-responsive genes; (v) Influencing rhizosphere microbes, favoring beneficial organisms for nutrient cycling and soil health (**Figure 8**). *Arthrospira* emerges as a promising biofertilizer for global saline agriculture, advancing plant growth, salt stress relief, and soil sustainability.

## Data availability statement

The data presented in the study are deposited in the NCBI Sequence Read Archive (SRA, https://www.ncbi.nlm.nih.gov/sra/) repository, accession number PRJNA995389 for RNA-seq and PRJNA1002101 for 16S rRNA sequencing. The names of the repository/repositories and accession number(s) can be found in the article/**Supplementary Material**.

## Author contributions

QX: Conceptualization, Data curation, Methodology, Visualization, Writing – original draft. TZ: Conceptualization, Data curation, Methodology, Validation, Writing – original draft. RZ: Data curation, Methodology, Software, Writing – original draft. YZ: Data curation, Methodology, Software, Writing – original draft. YD: Data curation, Methodology, Software, Writing – original draft. XL: Data curation, Methodology, Software, Writing – original draft. GL: Data curation, Methodology, Software, Writing – original draft. RH: Data curation, Methodology, Software, Writing – original draft. ST: Data curation, Methodology, Software, Writing – original draft. XM: Data curation, Methodology, Software, Writing – original draft. YL: Data curation, Methodology, Software, Writing – original draft. SL: Funding acquisition, Project administration, Resources, Supervision, Writing – review & editing. XFL: Funding acquisition, Project administration, Resources, Supervision, Writing – review & editing.
