# Supplementary material for: Arthrospira promotes plant growth and soil properties under high salinity environments
Source: Front Plant Sci. 2023 Dec 5;14:1293958. doi: 10.3389/fpls.2023.1293958 (PMC10728656; doi:10.3389/fpls.2023.1293958)
Supplement: Supplementary file 1 [file DataSheet_1.zip › Supplementary_Material.docx]

Supplementary Material

## Supplementary Tables

## Supplementary Table S1. Primers used for qRT-PCR in this study.

## Supplementary Table S2. The whole cell components of *Arthrospira*.

## Supplementary Table S3. Concentration of GG and Tre in *Arthrospira*.

## Supplementary Table S4. Basic information about the RNA-seq data used in this study.

## Supplementary Table S5. Shared DEGs identified in S_AP-S and CK-S.

## Supplementary Table S6. Analysis of Alpha diversity indices with or without *Arthrospira* application under salt stress.

##
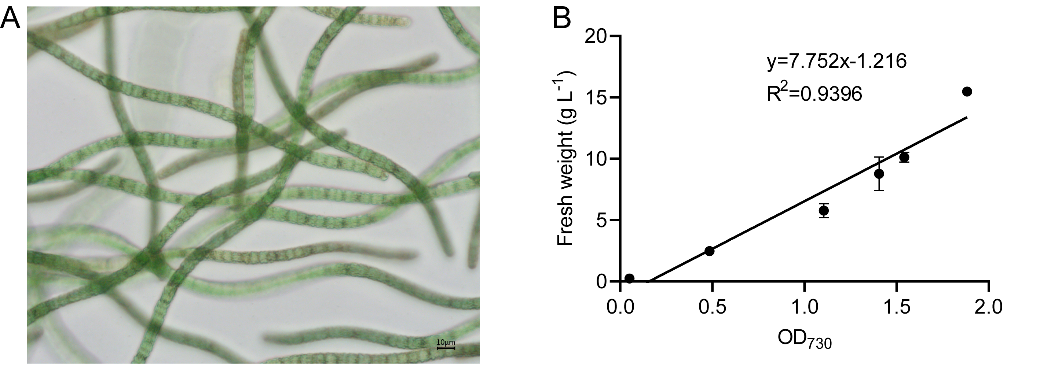
Supplementary Figures

# Supplementary Figure 1. Microscopy image and fresh weight calculation of *Arthrospira*. A, Microscopy image of *Arthrospira* in Zarrouk culture medium. B, The corresponding relation between OD_730_ and fresh weight of *Arthrospira*. Scar bars, 10 μm.

#
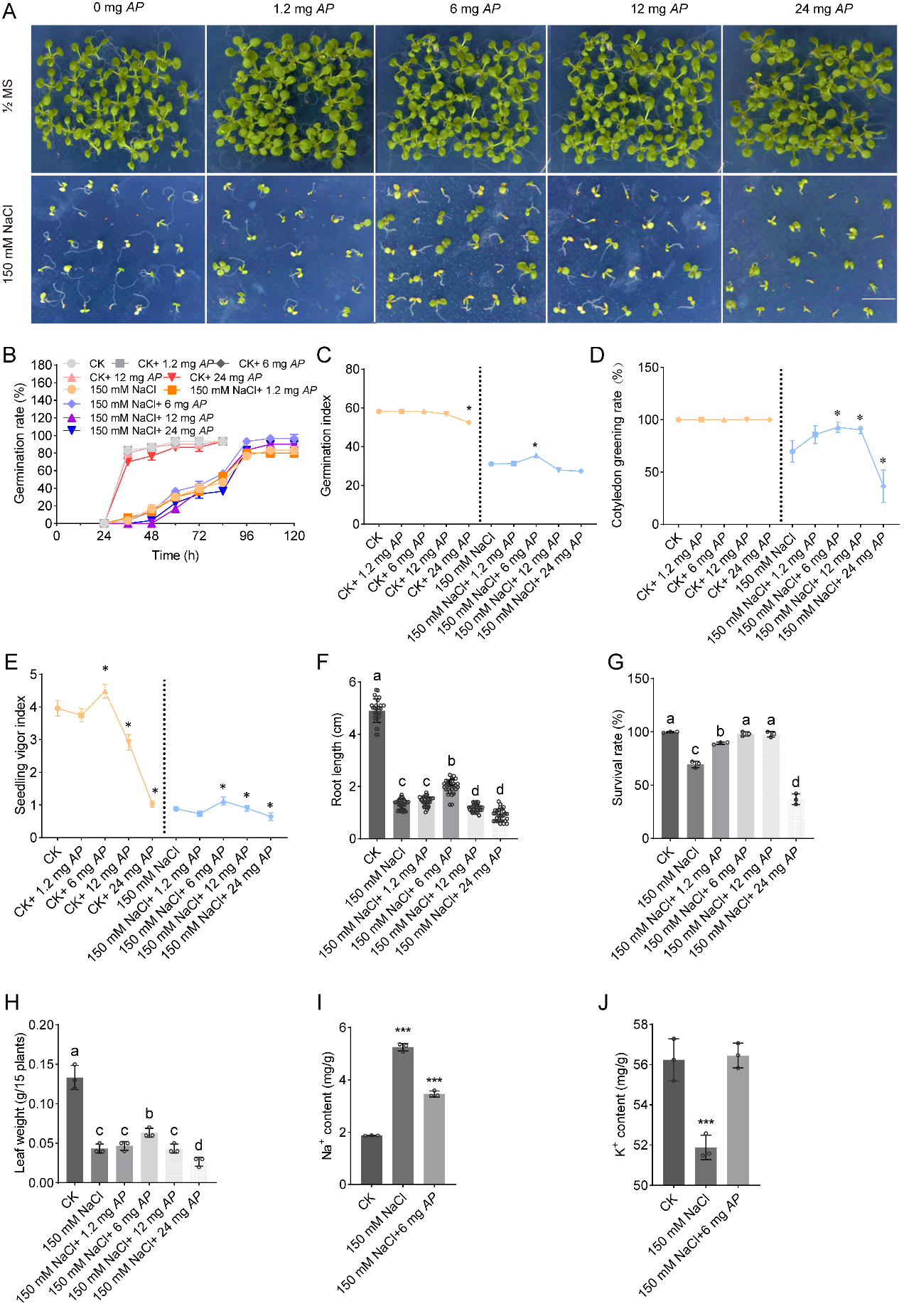
Supplementary Figure 2. The effect of *Arthrospira* treatment on the germination and growth of *Arabidopsis* grown on medium under salt stress. A, Phenotype of 7-day-old *Arabidopsis* seedlings grown on ½ MS medium with or without 150 mM NaCl with various concentrations of *Arthrospira*. B-E, Comparison of the germination rate (B), germination index (C), cotyledon greening rate (D) and seedling vigor index (E) of *Arabidopsis* in A. F-J, Comparison of the root length (F), survival rate (G), leaf weight (H), Na^+^ content (I) and K^+^ content (J) of 2-week-old *Arabidopsis* grown on 1/2 MS medium under 150 mM NaCl with various concentration of *Arthrospira*. *AP*, *Arthrospira platensis*. Scar bars, 1 cm.

#
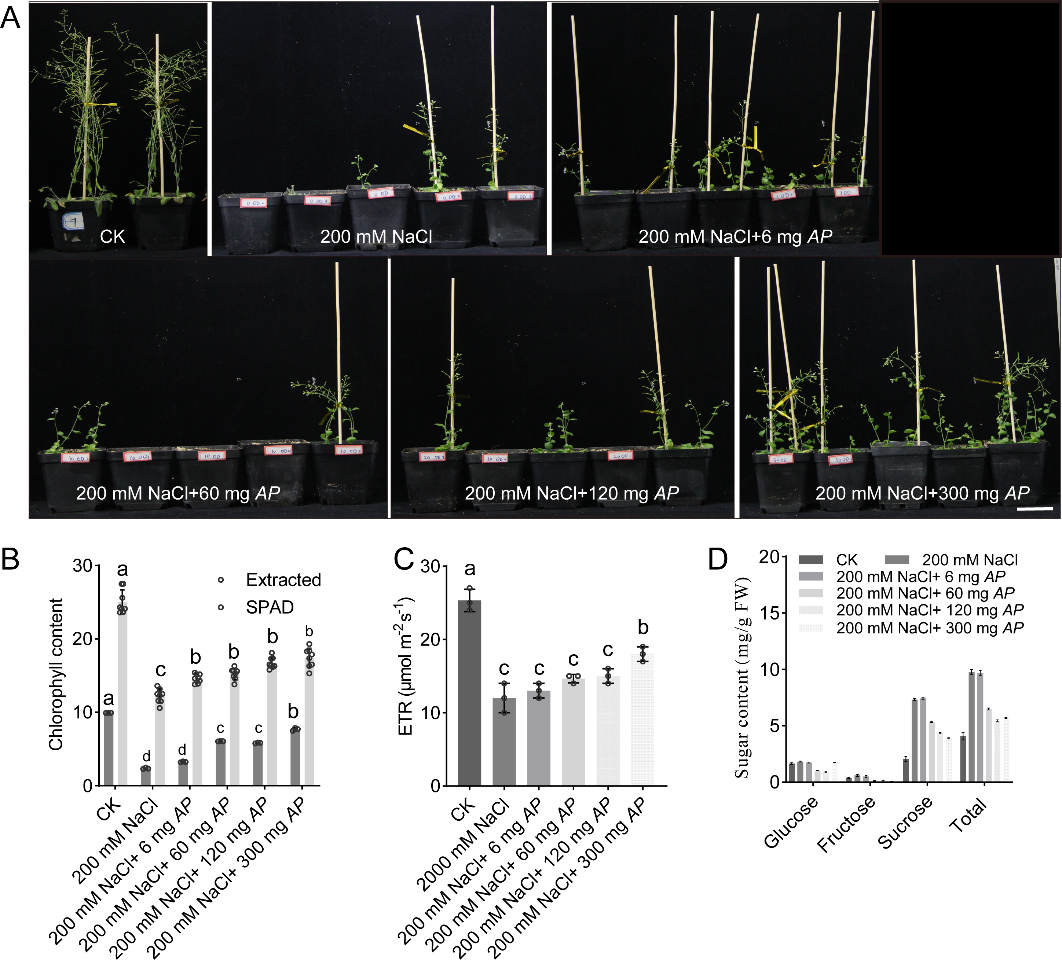
Supplementary Figure 3. The effect of *Arthrospira* treatment on the growth of *Arabidopsis* seedlings grown on soil under salt stress. A, Phenotypes of 4-day-old *Arabidopsis* seedlings grown on ½ MS medium were transferred to soil with 200 mM NaCl for 5 weeks containing various concentration of *Arthrospira*. B-D, Comparison of the chlorophyll content (B), relative photosynthetic electron transfer rate (ETR) (C) and sugar content (D) in A. *AP*, *Arthrospira platensis*. Scar bars, 5 cm.

#
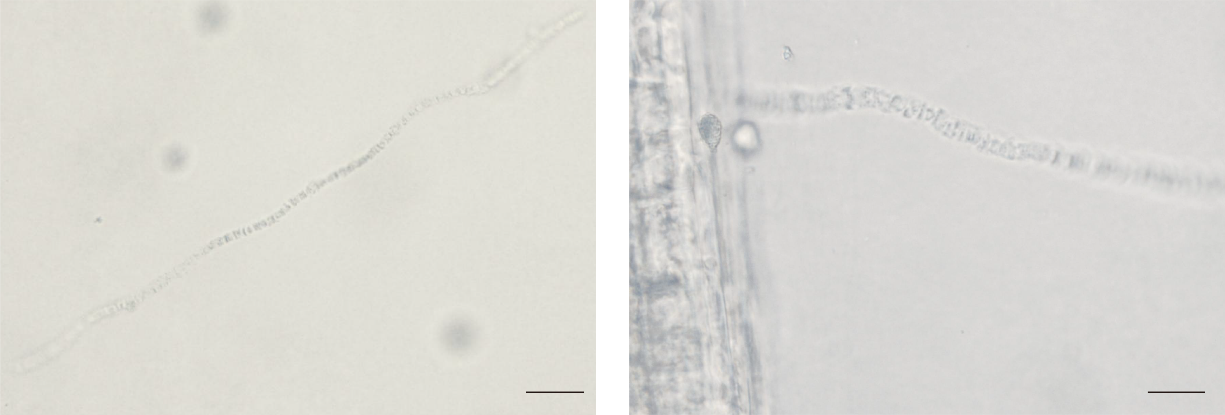
Supplementary Figure 4. Microscopy image of *Arthrospira* grown in ½ MS medium with 125 mM NaCl for 3 days. Scar bars, 10 μm.

#
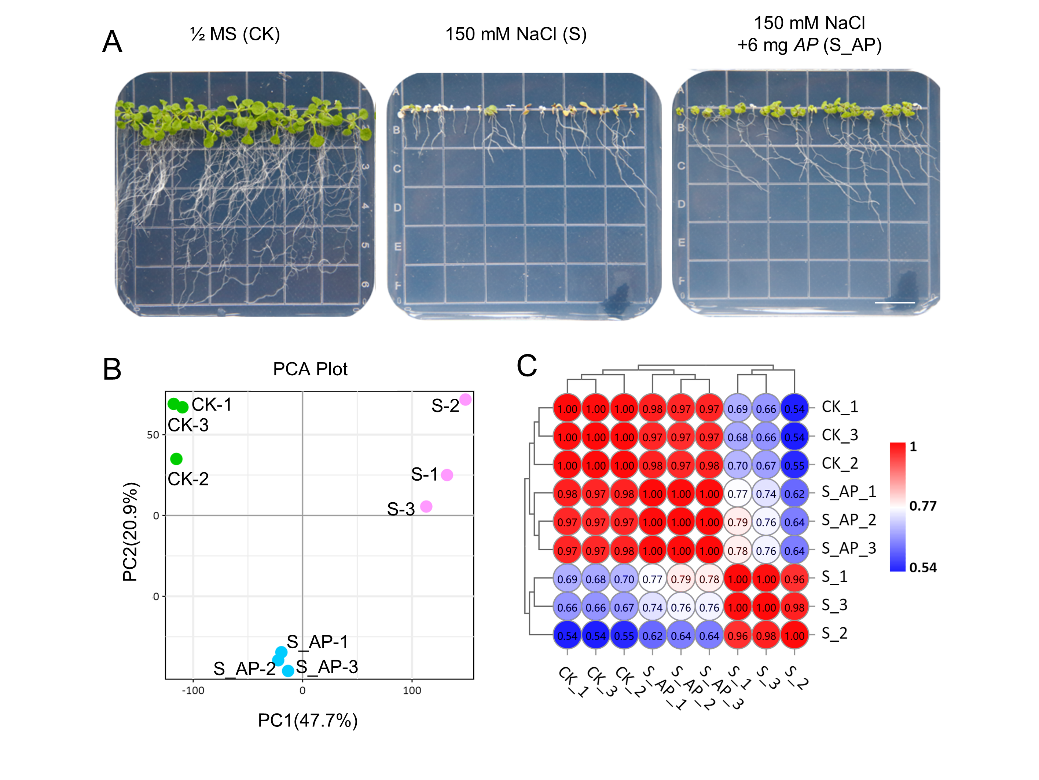
Supplementary Figure 5. PCA and correlation analysis of samples used in RNA-seq. A, Samples used in RNA-seq analysis. Pictures show the 21-day-old *Arabidopsis* seedlings grown on ½ MS medium with or without 150 mM NaCl with 6 mg *Arthrospira*. B, Principal component analysis (PCA) of unigene. C, Sample correlation analysis. CK represents *Arabidopsis* seedlings grown on normal ½ MS medium condition; S represents *Arabidopsis* seedlings grown on ½ MS medium containing 150 mM NaCl, and S_*AP* represents *Arabidopsis* seedlings grown on ½ MS medium containing 150 mM NaCl and 6 mg *Arthrospira*. *AP*, *Arthrospira platensis*.

#
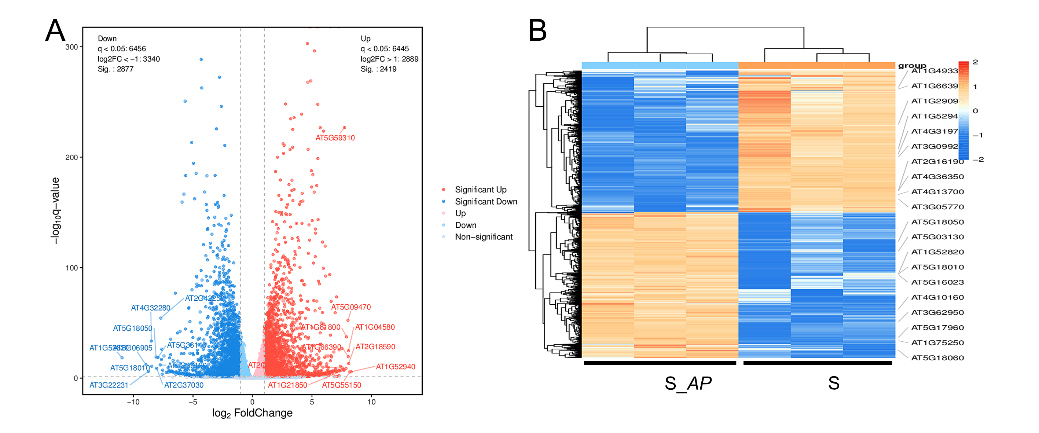
Supplementary Figure 6. Analysis of DEGs in *Arabidopsis* seedlings grown on ½ MS medium under 150 mM NaCl containing 6 mg *Arthrospira*. A, Valcano plot of log_2_-fold change values of DEGs in CK-S group. B, Hierarchical cluster analysis of the DEGs in S_*AP*-S group. The red and blue dots represent up- and down-regulated DEGs, respectively. The grey dots represent genes with no significant differences. S represents *Arabidopsis* seedlings grown on ½ MS medium containing 150 mM NaCl, and S_*AP* represents *Arabidopsis* seedlings grown on ½ MS medium containing 150 mM NaCl and 6 mg *Arthrospira*. *AP*, *Arthrospira platensis*.

#
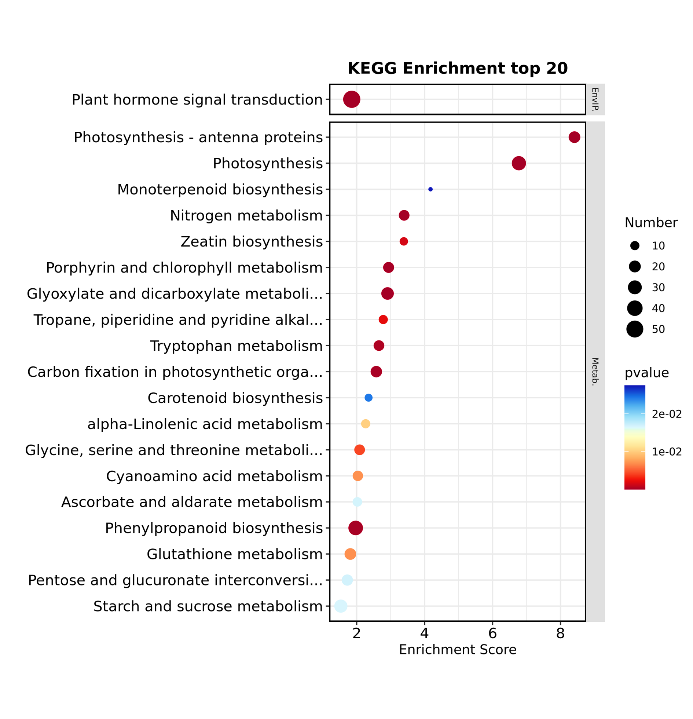
Supplementary Figure 7. Statistical analysis of KEGG pathway enrichment of all shared DEGs in *Arabidopsis* seedlings grown on ½MS medium supplemented with 150 mM NaCl containing 6 mg *Arthrospira*. KEGG, Kyoto encyclopedia of genes and genomes.

#
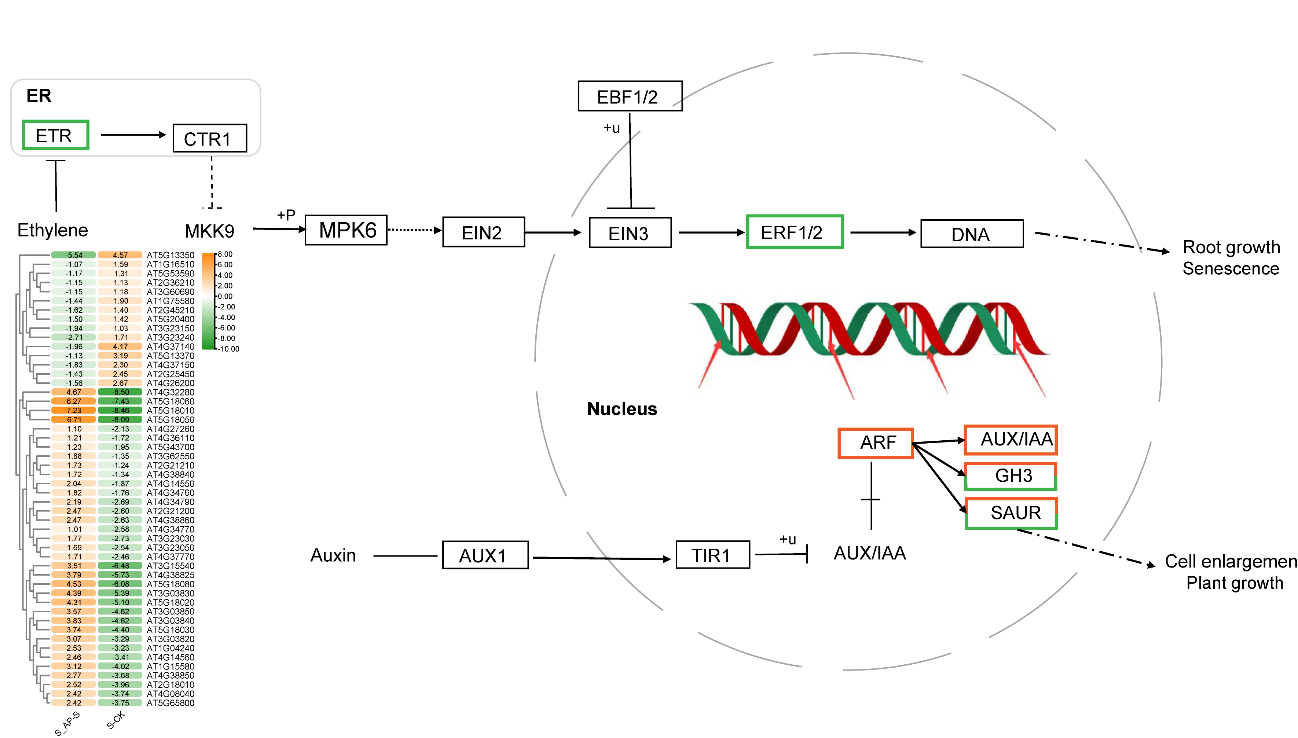
Supplementary Figure 8. Analysis of enriched KEGG pathways in plant hormone signal transduction. The orange and green scale indicate significantly up- and down-regulated genes, respectively (log_2_ |fold-change| ≥ 1). Arrows indicate enzyme reaction directions. AUX/IAA, auxin/indole-3-acetic acid; SAUR, small auxin upregulated RNA; GH3, Auxin-responsive GH3 family protein; ARF, auxin response factor; ETR, ethylene response receptor; ERF, ethylene response factor. '+u', '+p' and'-p' represented 'ubiquitination’, phosphorylation', and 'dephosphorylation', respectively.

#
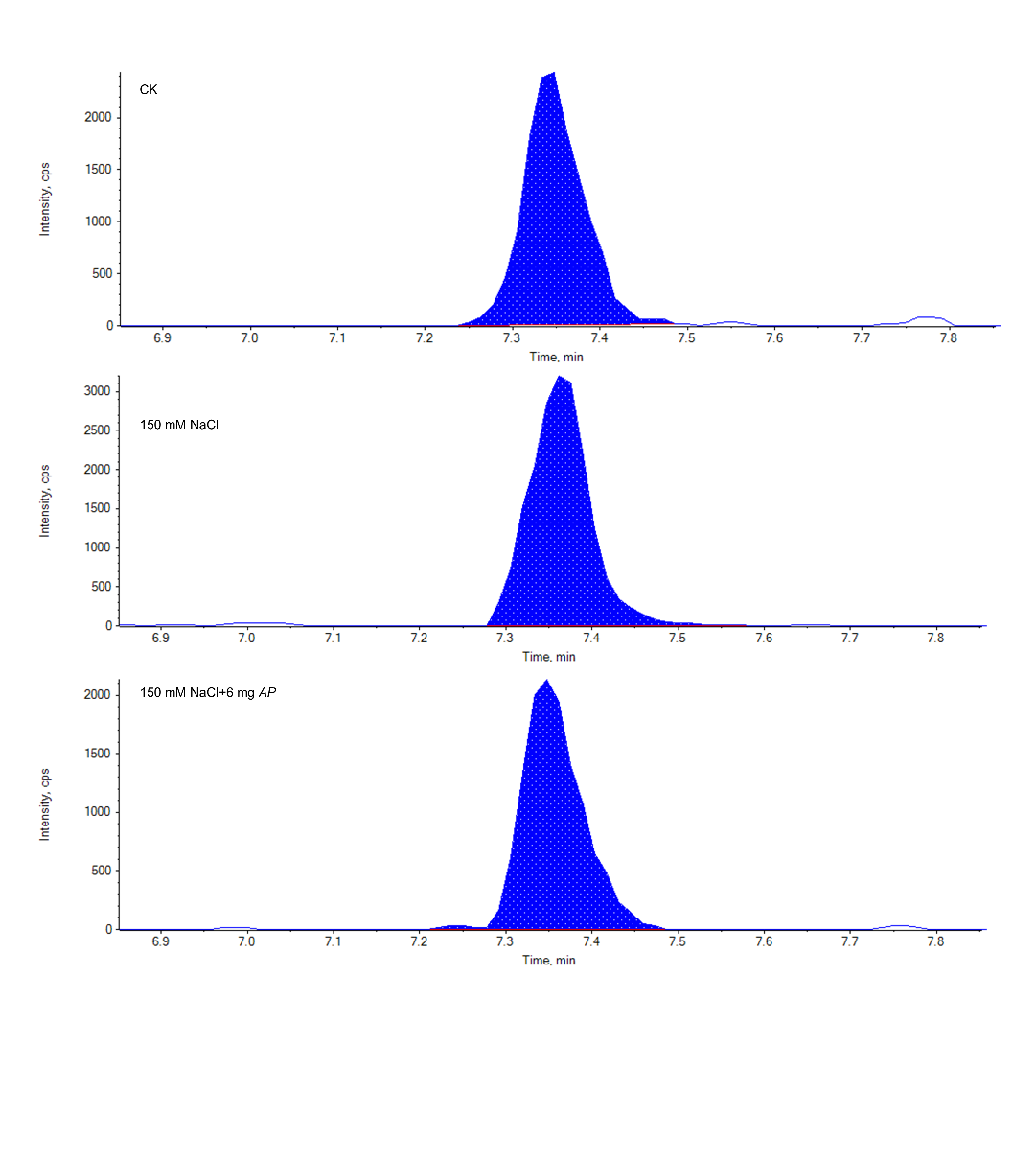
Supplementary Figure 9. Mass spectrometry peak chromatogram for ABA. The peak elution time for ABA is about 7.35 minutes. The content is calculated based on the peak area.

#
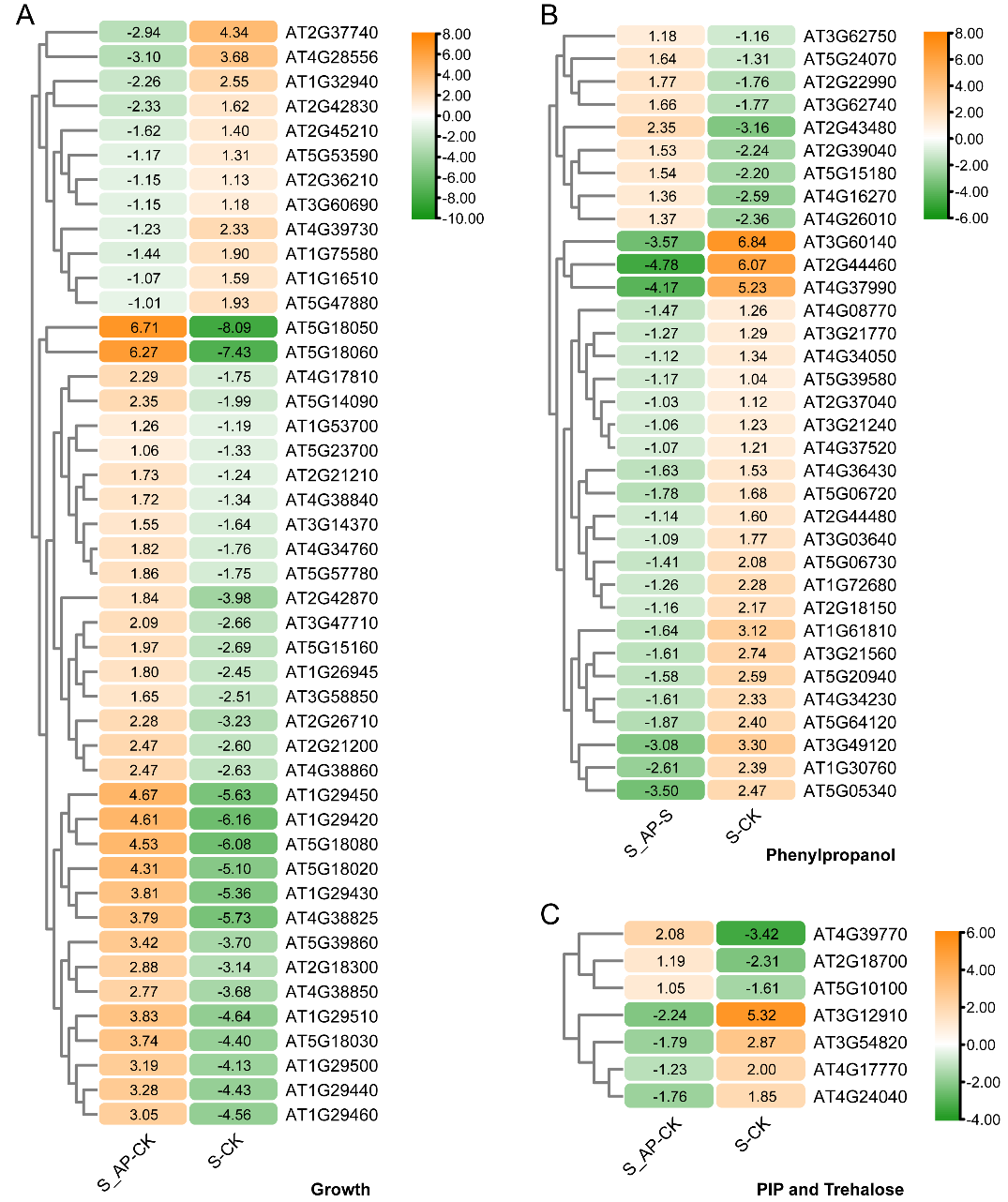
Supplementary Figure 10. Hierarchical cluster analysis of the shared DEGs in S-Ck and S_*AP*-S. A-C, Heatmap analysis of genes associated with growth (A), phenylpropanol (B) and PIP and Trehalose (C) in *Arabidopsis* seedlings grown on ½ MS medium under 150 mM NaCl containing 6 mg *Arthrospira*. The color scale represents the log_2_-fold change values (S_*AP*-S or S-CK). The orange and green scale indicate significantly up- and down-regulated genes, respectively (log2 |fold-change| ≥ 1). CK represents *Arabidopsis* seedlings grown on normal ½ MS medium condition; S represents *Arabidopsis* seedlings grown on ½ MS medium containing 150 mM NaCl, and S_*AP* represents *Arabidopsis* seedlings grown on ½ MS medium containing 150 mM NaCl and 6 mg *Arthrospira*. *AP*, *Arthrospira platensis.*

#
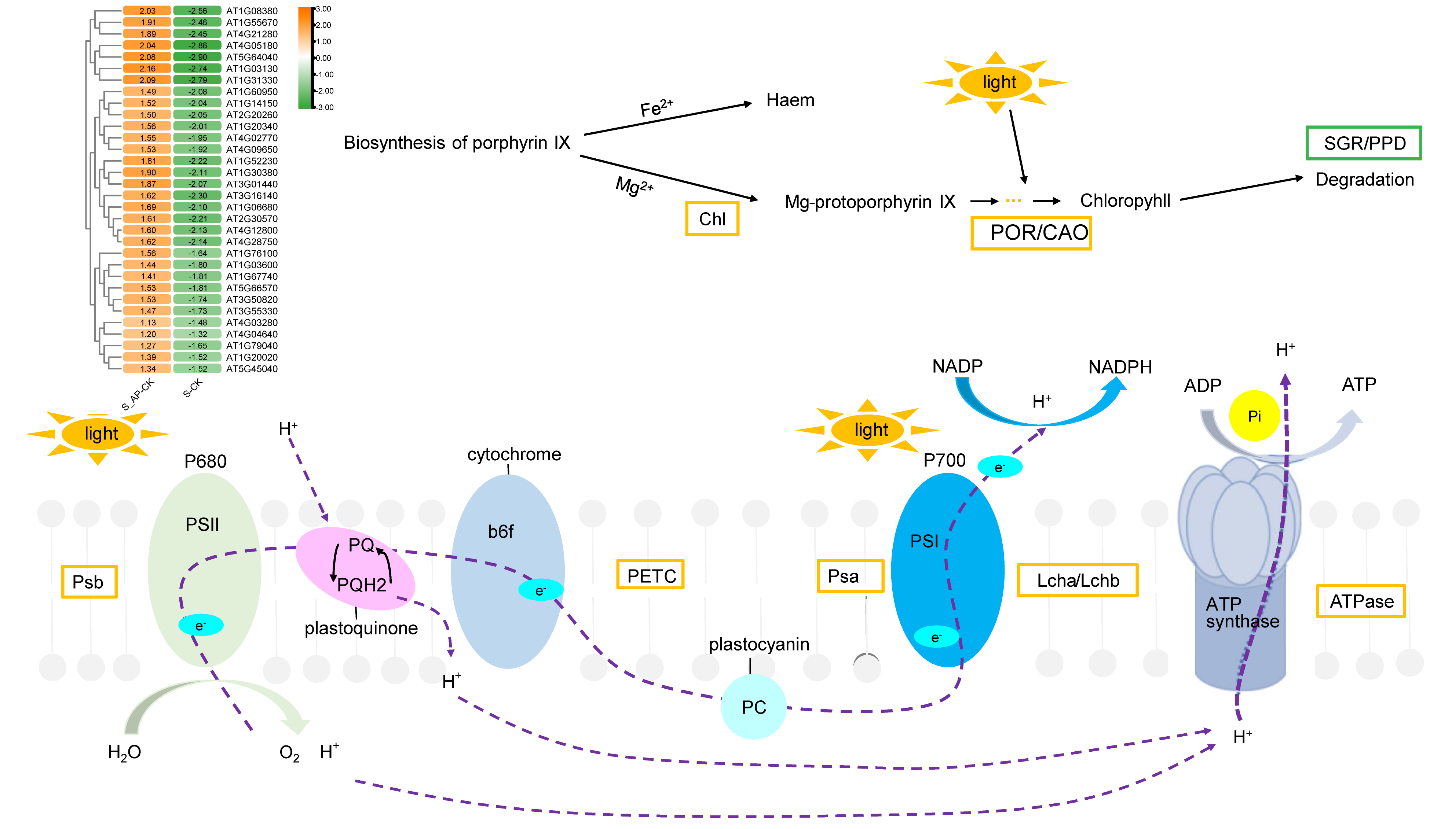
Supplementary Figure 11. Analysis of plant cholorophyll metabolism and photosynthesis of enriched KEGG pathway. The orange and green scale indicate significantly up- and down-regulated genes, respectively (log_2_ |fold-change| ≥ 1). Arrows indicate enzyme reaction directions. Chl, magnesium chelatase subunit; POR, protochlorophyllide oxidoreductase; CAO, chlorophyllide a oxygenase; SGR, stay-green, PPD, pyruvate orthophosphate dikinase; PSII, photosystem II (Psb); PSI, photosystem I (Psa); antenna proteins (Lhca); PETC, photosynthetic electron transfer C; F-type ATPases proteins (ATPase-gamma, delta).

#
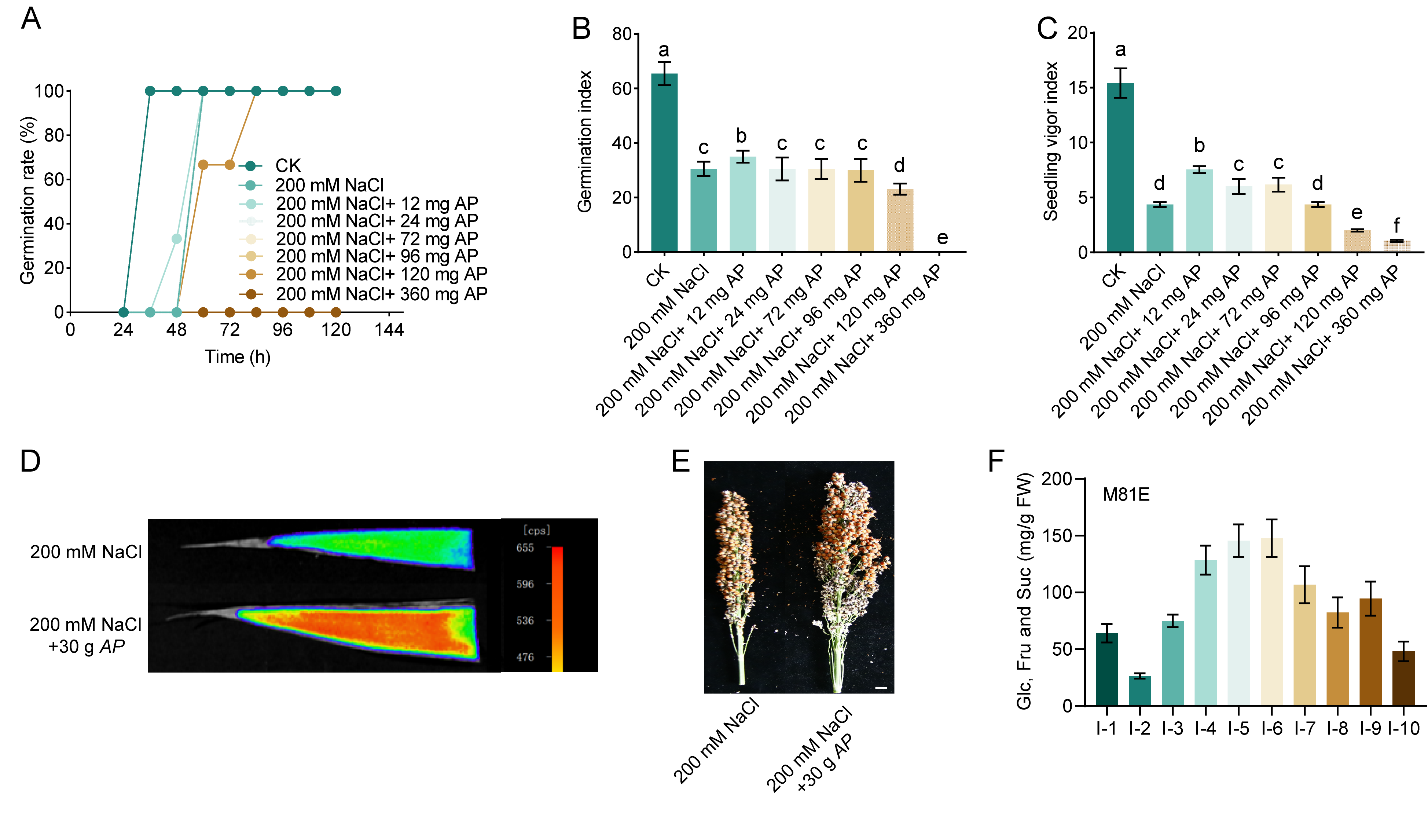
Supplementary Figure 12. The effect of *Arthrospira* treatment on the germination and growth of sweet sorghum under salt stress. A-C, Comparison of the germination rate (A), germination index (B) and seedling vigor index (C) for sweet sorghum grown on filter paper with 200 mM NaCl containing various concentration of *Arthrospira*. D, Delayed fluorescence of sweet sorghum under 200 mM NaCl condition with or without *Arthrospira* treatment. E, Phenotype of sorghum panicles under 200 mM NaCl treatment with or without 30 g *Arthrospira*. F, Concentration of soluble sugars (glucose, fructose and sucrose) during flowering stage at about 100 days. The youngest internode is positioned at the top and the oldest internode at the bottom, starting from the internode I-1 to internode I-10. *AP*, *Arthrospira platensis.* Scar bars, 1 cm.

#
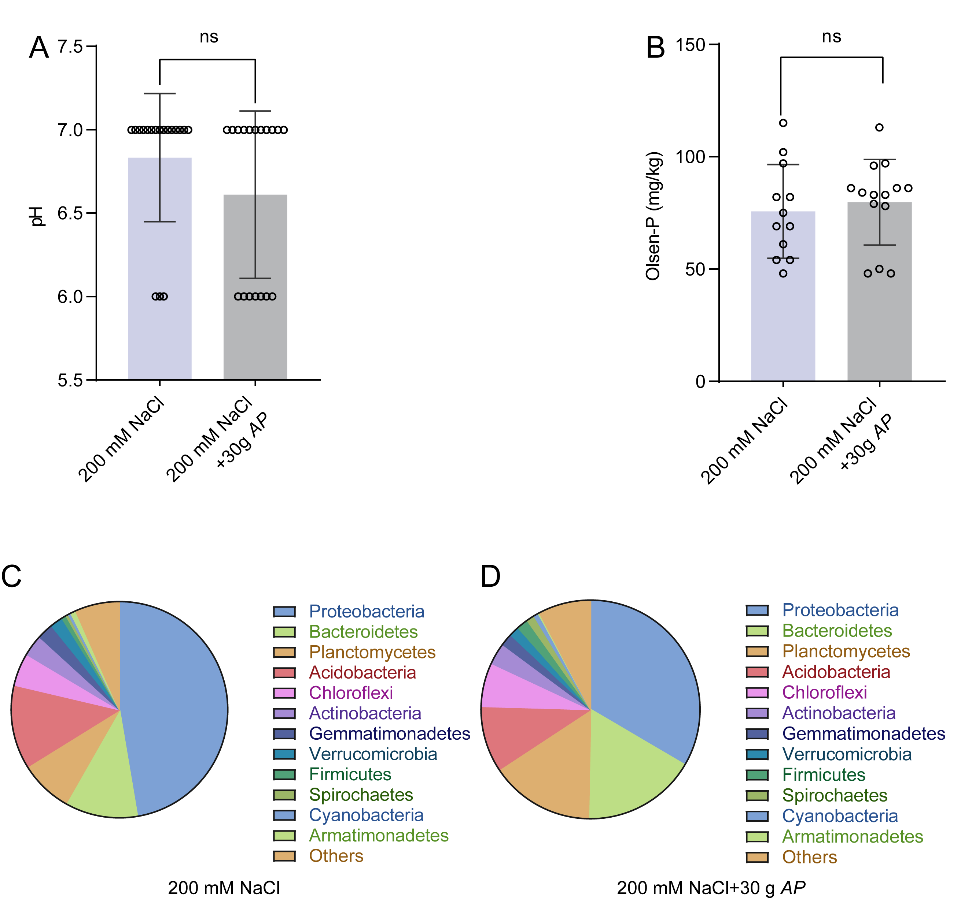


# Supplementary Figure 13. *Arthrospira* alters the soil chemical properties and structure of rhizosphere. A-B, Soil pH (A) and Olsen-P (C) in soil where sorghum grown with or without 30 g *Arthrospira*. C-D, Pie charts represent the phylum-level distribution of rhizosphere bacterial communities without (C) and with (D) *Arthrospira* treatment. The twelve most abundant bacterial phyla with relative abundance >0.5% were listed, while "Others" indicates the remaining phyla with relative abundance <0.5%.
